# NF-κB signaling in osteoarthritis: integrating mechanical stress, innate immunity, and cartilage degeneration

**DOI:** 10.3389/fimmu.2026.1842443

**Published:** 2026-06-05

**Authors:** Peng Wan, Yimin Zheng

**Affiliations:** 1 Department of Orthopedics, Shengzhou People’s Hospital (Shengzhou Branch of the First Affiliated Hospital of Zhejiang University School of Medicine, the Shengzhou Hospital of Shaoxing University), Shengzhou, Zhejiang, China; 2 Department of Hand and Foot Surgery, The Xinchang Affiliated Hospital, Shaoxing University, Xinchang, Zhejiang, China

**Keywords:** cartilage degeneration, inflammation, innate immunity, mechanotransduction, NF-κB signaling, osteoarthritis

## Abstract

Osteoarthritis (OA) is a complex and multifactorial joint disease that was traditionally regarded as a consequence of mechanical wear and tear, but is now increasingly recognized as a disorder driven by chronic low-grade inflammation and dysregulated stress responses. Nuclear factor-κB (NF-κB) has emerged as a pivotal regulator in OA; however, its role extends well beyond that of a linear inflammatory pathway. In this review, we synthesize recent evidence supporting NF-κB as an important integrative signaling node through which mechanical stress, innate immune activation, and metabolic cues converge to influence joint degeneration. We discuss how mechanotransduction mediated by ion channels, mitochondrial dysfunction-associated DNA sensing, and danger-associated molecular patterns converge on NF-κB activation, thereby establishing feed-forward inflammatory circuits. Downstream, NF-κB-associated signaling is closely involved in extracellular matrix degradation, chondrocyte fate decisions—including senescence, apoptosis, and ferroptosis—synovial inflammation and fibrosis, immune microenvironment remodeling, and subchondral bone alterations. Importantly, the biological consequences of NF-κB signaling are highly context dependent and are shaped by the source, intensity, and duration of upstream stimuli, as well as by the metabolic and aging status of joint cells. Finally, we summarize emerging therapeutic strategies targeting NF-κB-associated networks at multiple hierarchical levels and highlight the importance of mechanism-based patient stratification and rational combination therapies. By positioning NF-κB as a hub of signaling integration, this review provides a unifying framework for understanding OA pathogenesis and suggests new directions for precision interventions aimed at restoring joint homeostasis.

## Highlights

- NF-κB functions as an important integrative signaling node linking mechanical stress, innate immune sensing, and metabolic inflammation in osteoarthritis.
- Context-dependent NF-κB signaling shapes cartilage matrix remodeling, chondrocyte stress responses, and immune microenvironment changes in OA.
- Mechanism-based stratification and network-oriented combination strategies may help advance precision treatment in OA.

## 1 Introduction

Osteoarthritis (OA) is the most prevalent degenerative joint disorder worldwide and a leading cause of pain, disability, and reduced quality of life in aging populations (1). Traditionally, OA has been regarded as a purely degenerative disease driven by mechanical wear and tear of articular cartilage (2). However, accumulating experimental and clinical evidence has fundamentally revised this view. OA is now recognized as a complex, whole-joint disease characterized by chronic low-grade inflammation, dysregulated mechanotransduction, and maladaptive tissue remodeling involving cartilage, synovium, subchondral bone, and immune cells (3). This paradigm shift has placed inflammatory signaling networks—rather than passive cartilage erosion—at the center of OA pathogenesis.

The pathological hallmark of OA lies in the dynamic interplay between degenerative and inflammatory processes (4–6). Articular cartilage degeneration does not occur in isolation, but is tightly linked to synovial inflammation, subchondral bone remodeling, and changes in the joint immune microenvironment.modelingMechanical overload, aging-associated cellular stress, metabolic dysfunction, and microinjury collectively generate danger-associated molecular patterns (DAMPs), inflammatory mediators, and oxidative stress signals (7, 8). These stimuli converge to drive persistent activation of inflammatory transcriptional programs that, in turn, accelerate extracellular matrix (ECM) degradation, chondrocyte dysfunction, and joint structural failure. Within this complex signaling landscape, NF-κB has emerged as an important inflammatory mediator and integrative signaling node in OA signaling (9). NF-κB signaling is well positioned to function as an integrative node because it receives and interprets a broad spectrum of upstream inputs signaling—including mechanical stress, ion channel–mediated calcium flux, innate immune sensing, cytokine stimulation, and metabolic stress—and translates them into coordinated transcriptional responses (10, 11). Activation of NF-κB promotes the expression of pro-inflammatory cytokines, chemokines, matrix-degrading enzymes, and cell fate regulators, thereby linking mechanical and inflammatory cues to tissue degeneration (12). Importantly, NF-κB activation in OA is neither spatially uniform nor temporally transient. Instead, it displays context-dependent patterns shaped by the intensity, duration, and cellular origin of upstream stimuli (13). In chondrocytes, sustained NF-κB signaling disrupts anabolic–catabolic balance, accelerates ECM breakdown, and drives cellular senescence, apoptosis, or ferroptosis (14). In synovial fibroblasts and macrophages, NF-κB activation contributes to synovitis, fibrosis, and immune cell recruitment (15). In subchondral bone, NF-κB contributes to aberrant bone remodeling and osteoclastogenesis (16). These compartment-specific effects suggest that NF-κB functions not only as a downstream inflammatory effector but also as an important mediator of multicellular and multi-tissue crosstalk within the osteoarthritic joint.

In this review, we synthesize recent advances that position NF-κB as an important integrative signaling node linking mechanical stress, innate immune activation, and cartilage degeneration in OAsignaling. Rather than treating NF-κB as a linear inflammatory pathway, we emphasize its role as an integrative network node that may influence disease progression through feed-forward amplification loops and pathway crosstalkforward. Specifically, we organize the review around a hub-and-spoke framework centered on NF-κB signaling. First, we examine upstream triggers that initiate or sustain NF-κB activation in OA, with particular emphasis on mechanical stress–induced mechanotransduction, ion channel–mediated calcium signaling, and innate immune sensors such as pattern recognition receptors and cytosolic DNA-sensing pathways. We then discuss how distinct modes of NF-κB-associated signaling relate to downstream pathological processes, including inflammatory gene expression, extracellular matrix (ECM) remodeling, chondrocyte fate changes(senescence, apoptosis, and ferroptosis), synovial inflammation and fibrosis, and immune microenvironment remodeling through macrophage polarization and chemokine signaling. Finally, we integrate these mechanistic insights with emerging therapeutic strategies that target NF-κB itself or its upstream and downstream regulatory nodes. By mapping pharmacological agents, physical interventions, and natural compounds onto the NF-κB-centered signaling network, we aim to highlight actionable intervention points and identify opportunities for rational combination therapies. Through this systems-level perspective, the review seeks to provide a unifying conceptual framework for understanding OA as a disorder of dysregulated signaling integration rather than isolated tissue degeneration, thereby informing future translational and clinical efforts.

## 2 NF-κB signaling in osteoarthritis: pathway architecture and activation patterns

NF-κB signaling represents one of the most extensively studied inflammatory pathways; however, its role in osteoarthritis extends well beyond a simple pro-inflammatory cascade. In OA, NF-κB functions as a dynamic and context-dependent signaling mediator whose activation patterns, tissue specificity, and pathway crosstalk influence disease outcomes signaling. Understanding the architectural features of NF-κB signaling and its activation modes is therefore essential for interpreting how diverse upstream stimuli converge on joint degeneration.

### 2.1 Canonical versus non-canonical NF-κB signaling: structural basis and functional dynamics

NF-κB signaling is broadly divided into canonical and non-canonical pathways, which differ in upstream triggers, molecular components, and biological outputs (17, 18). The canonical NF-κB pathway is predominantly activated by pro-inflammatory cytokines, mechanical stress–induced signals, pattern recognition receptors, and cellular stress cues. These stimuli converge on the IκB kinase (IKK) complex, composed of IKKα, IKKβ, and the regulatory subunit NEMO (IKKγ). Activation of the IKK complex leads to phosphorylation and proteasomal degradation of IκB proteins, thereby releasing NF-κB dimers—most commonly p65 (RelA)/p50—for nuclear translocation and transcriptional activation (19). In contrast, the non-canonical NF-κB pathway is typically engaged by a restricted subset of stimuli and relies on NF-κB–inducing kinase (NIK)–dependent processing of p100 into p52, resulting in p52/RelB nuclear signaling (20). Although the non-canonical pathway has been less extensively characterized in OA, emerging evidence suggests that it may contribute to chronic inflammatory remodeling, immune cell differentiation, and bone metabolism under specific conditions. Crucially, NF-κB signaling outcomes are not determined solely by pathway identity but by the dynamics of activation, including signal amplitude, duration, and temporal oscillation. Transient or low-level NF-κB activation may support adaptive stress responses or tissue homeostasis, whereas sustained or repetitive activation drives pathological programs such as chronic inflammation, cellular senescence, apoptosis, or regulated cell death (21). In OA, repeated mechanical overload, persistent inflammatory cues, and unresolved cellular stress favor prolonged NF-κB activity, thereby locking joint tissues into a self-reinforcing degenerative state (13). This dynamic perspective helps explain why NF-κB can mediate both physiological and pathological responses depending on context.

### 2.2 Tissue-specific NF-κB activation patterns across the osteoarthritic joint

OA is increasingly recognized as a disease of the entire joint organ, and NF-κB signaling exhibits distinct activation patterns across different tissue compartments (22). In articular cartilage, NF-κB is closely involved in shifting chondrocytes from a homeostatic, anabolic phenotype toward a catabolic and degenerative state toward. Under physiological conditions, tightly regulated NF-κB activity participates in stress adaptation and survival. However, in OA, sustained NF-κB activation disrupts extracellular matrix homeostasis by promoting matrix-degrading programs and suppressing anabolic signaling (23). Beyond matrix regulation, NF-κB critically influences chondrocyte fate decisions, driving cellular senescence, apoptosis, or ferroptosis depending on the nature and persistence of upstream stressors (24, 25). These processes collectively reduce the reparative capacity of cartilage and accelerate structural breakdown. In the synovium, NF-κB signaling is a major driver of inflammatory activation and fibrotic remodeling. Synovial fibroblasts and infiltrating immune cells respond to mechanical stress, DAMPs, and cytokines with robust NF-κB activation, leading to the production of pro-inflammatory mediators and chemokines that amplify immune cell recruitment. Prolonged NF-κB signaling in the synovium contributes not only to chronic synovitis but also to pathological fibrosis, which exacerbates joint stiffness and pain. Importantly, synovial NF-κB activity serves as a key source of inflammatory signals that propagate damage to adjacent cartilage and subchondral bone (26, 27). In subchondral bone, NF-κB signaling regulates the balance between bone resorption and formation Activation of NF-κB in osteoclast precursors promotes osteoclastogenesis and bone resorption, while dysregulated signaling in osteoblast-lineage cells alters bone formation and mineralization. These changes lead to aberrant subchondral bone remodeling, which in turn modifies mechanical load distribution across the joint and feeds back to exacerbate cartilage degeneration. Thus, NF-κB acts as a molecular conduit linking inflammatory signaling to biomechanical alterations in OA (28).

### 2.3 NF-κB as a signaling hub: network crosstalk with MAPK, PI3K/AKT, mTOR, and Nrf2 pathways

A defining feature of NF-κB signaling in OA is its extensive crosstalk with other major intracellular pathways, reinforcing its role as an important integrative node rather than an isolated axis signaling (29). NF-κB signaling intersects with mitogen-activated protein kinase (MAPK) pathways to coordinate inflammatory gene expression and stress responses, particularly under conditions of mechanical overload or cytokine stimulation (30). Similarly, the PI3K/AKT pathway modulates NF-κB activity by influencing IKK activation (31), transcriptional cofactor recruitment, and cell survival programs, thereby shaping the balance between inflammation and apoptosis. The mechanistic target of rapamycin (mTOR) pathway further integrates metabolic status with NF-κB–driven inflammatory outputs. Dysregulated mTOR signaling in OA can synergize with NF-κB to promote catabolic metabolism, suppress autophagy, and exacerbate chondrocyte dysfunction (32). Conversely, inhibition of mTOR has been shown to attenuate NF-κB–mediated inflammatory damage in experimental models. Counterbalancing these pro-inflammatory networks, the nuclear factor erythroid 2–related factor 2 (Nrf2) pathway acts as a key antioxidant and cytoprotective regulator that antagonizes NF-κB signaling (33). Crosstalk between Nrf2 and NF-κB determines the cellular redox state, inflammatory tone, and susceptibility to oxidative stress–induced damage. In OA, impaired Nrf2 activity permits unchecked NF-κB activation, whereas restoration of Nrf2 signaling can partially reestablish redox and inflammatory balance (34). This dense network of pathway interactions places NF-κB within a multilayered signaling web center signaling that integrates mechanical cues, immune activation, metabolic status, and oxidative stress. Importantly, many emerging therapeutic interventions in OA exert their protective effects not by directly inhibiting NF-κB DNA binding, but by modulating these interconnected pathways. Recognizing NF-κB as a hub within this signaling network provides a conceptual foundation for understanding why diverse pharmacological agents, physical therapies, and natural compounds converge on NF-κB–associated nodes to restore joint homeostasis.

### 2.4 Dynamic and context-dependent NF-κB signaling: feedback control, pathway heterogeneity, and systems-level interpretation

A major challenge in interpreting NF-κB signaling in osteoarthritis is that it is often described as a binary inflammatory pathway, whereas its biological behavior is inherently dynamic, heterogeneous, and context dependent (35). In reality, NF-κB activity is not simply determined by whether the pathway is activated, but by how strongly, how long, in which cell type, and under what pre-existing cellular state the signal is propagated. This distinction is particularly important in OA, where chronic low-grade stress, recurrent biomechanical injury, senescence-associated inflammation, and metabolic dysfunction collectively shape signaling persistence rather than transient activation alone (36, 37). From this perspective, NF-κB should be viewed not as a uniform output node, but as a dynamic regulatory system whose downstream consequences are encoded by activation kinetics, feedback restraint, and tissue context.

At the molecular level, NF-κB signaling is governed by multiple layers of negative feedback and pathway termination that are often underappreciated in simplified mechanistic models (38, 39). Canonical NF-κB activation is normally constrained by IκBα resynthesis, ubiquitin-editing regulators such as A20/TNFAIP3, receptor desensitization, phosphatase-dependent deactivation, and restoration of organelle homeostasis after cellular stress (40–42). Under physiological conditions, these feedback modules help limit the duration and amplitude of NF-κB signaling, allowing stress adaptation without persistent inflammatory commitment. In OA, however, repeated exposure to mechanical overload, unresolved DAMP signaling, mitochondrial dysfunction, oxidative stress, and the senescence-associated secretory phenotype may progressively weaken these termination mechanisms (43). As a result, transient adaptive NF-κB responses can be converted into sustained and self-reinforcing inflammatory programs. Thus, OA progression may reflect not only excessive pathway activation, but also failure of feedback control and incomplete signal resolution. This dynamic view also helps explain why dose and duration of upstream stimuli are critical determinants of NF-κB output. Mechanical or inflammatory inputs do not elicit identical biological consequences across all intensity ranges. Low-level or transient NF-κB activation may support cell survival, stress buffering, antioxidant defense, and short-term tissue adaptation, whereas repetitive or sustained activation drives catabolic gene expression, inflammatory amplification, fibrosis, and regulated cell death (44, 45). In this regard, NF-κB signaling in OA is better conceptualized through a dose–duration–state framework, in which pathway output is shaped by the magnitude of stimulation, the temporal persistence of signaling, and the intrinsic condition of the responding cell. This systems-level interpretation provides a more coherent explanation for why moderate mechanical loading may preserve joint homeostasis, whereas chronic overload promotes degeneration.

Equally important, NF-κB signaling is not uniform across joint compartments. In chondrocytes, NF-κB primarily controls the balance between matrix maintenance and catabolic reprogramming while also influencing senescence, apoptosis, and ferroptosis (46, 47). In synovial fibroblasts, it preferentially drives inflammatory mediator release, fibroblast activation, and tissue fibrosis. In synovial macrophages, NF-κB shapes polarization states, cytokine production, and chemokine-dependent immune recruitment (48, 49). In subchondral bone-associated cells, NF-κB contributes to osteoclastogenic signaling and remodeling-associated alterations in bone turnover. These compartment-specific outputs indicate that NF-κB signaling is shared but not equivalent across the osteoarthritic joint. The same upstream trigger may therefore generate distinct pathological consequences depending on the cellular and spatial context in which it is interpreted.

An additional layer of complexity arises from pathway heterogeneity at the single-cell level. Even within the same tissue compartment, cells are unlikely to respond identically to equivalent mechanical or inflammatory stimuli. Emerging single-cell transcriptomic, epigenomic, and spatial profiling studies increasingly support the concept that OA tissues are composed of heterogeneous cellular states rather than homogeneous cell populations. Although direct single-cell resolution of NF-κB signaling dynamics remains limited, these datasets strongly suggest that inflammatory competence, stress-response capacity, chromatin accessibility, and catabolic susceptibility vary substantially across chondrocyte, fibroblast, macrophage, and bone-associated cell subsets. Accordingly, future integration of single-cell multi-omics with spatial mapping and phospho-signaling analysis will be essential for defining how NF-κB pathway heterogeneity contributes to disease progression and therapeutic vulnerability in OA. Taken together, these considerations argue that NF-κB should be interpreted as a dynamically regulated and spatially heterogeneous signaling network rather than a single linear inflammatory cascade.

## 3 Mechanical stress as an upstream driver of NF-κB in osteoarthritis

Mechanical loading is an indispensable physiological stimulus for joint homeostasis, yet it is also a dominant pathogenic force in osteoarthritis when exceeding adaptive thresholds. A defining feature of OA is the conversion of biomechanical cues into inflammatory transcriptional programs, a process termed mechanotransduction. NF-κB signaling occupies an important position in this conversion, acting as a molecular mediator that helps translate mechanical inputs into context-dependent inflammatory or protective outcomes signaling. Importantly, the biological consequences of mechanical stress are not binary but are dictated by its magnitude, duration, and temporal pattern, giving rise to a dose-dependent framework of NF-κB activation (50, 51).

### 3.1 Mechanotransduction: from mechanical overload to inflammatory transcription

Mechanical overload disrupts the structural and biochemical equilibrium of joint tissues, generating abnormal strain, shear stress, and hydrostatic pressure. These forces are sensed by mechanosensitive ion channels and structural organelles, triggering intracellular calcium influx, cytoskeletal remodeling, and activation of inflammatory signaling cascades. Among these pathways, NF-κB serves as a primary transcriptional effector linking mechanical stress to inflammation, inflammasome activation, and tissue remodeling.

#### 3.1.1 Piezo1-mediated NF-κB/NLRP3 axis in synovitis and fibrosis

**Figure 1:**
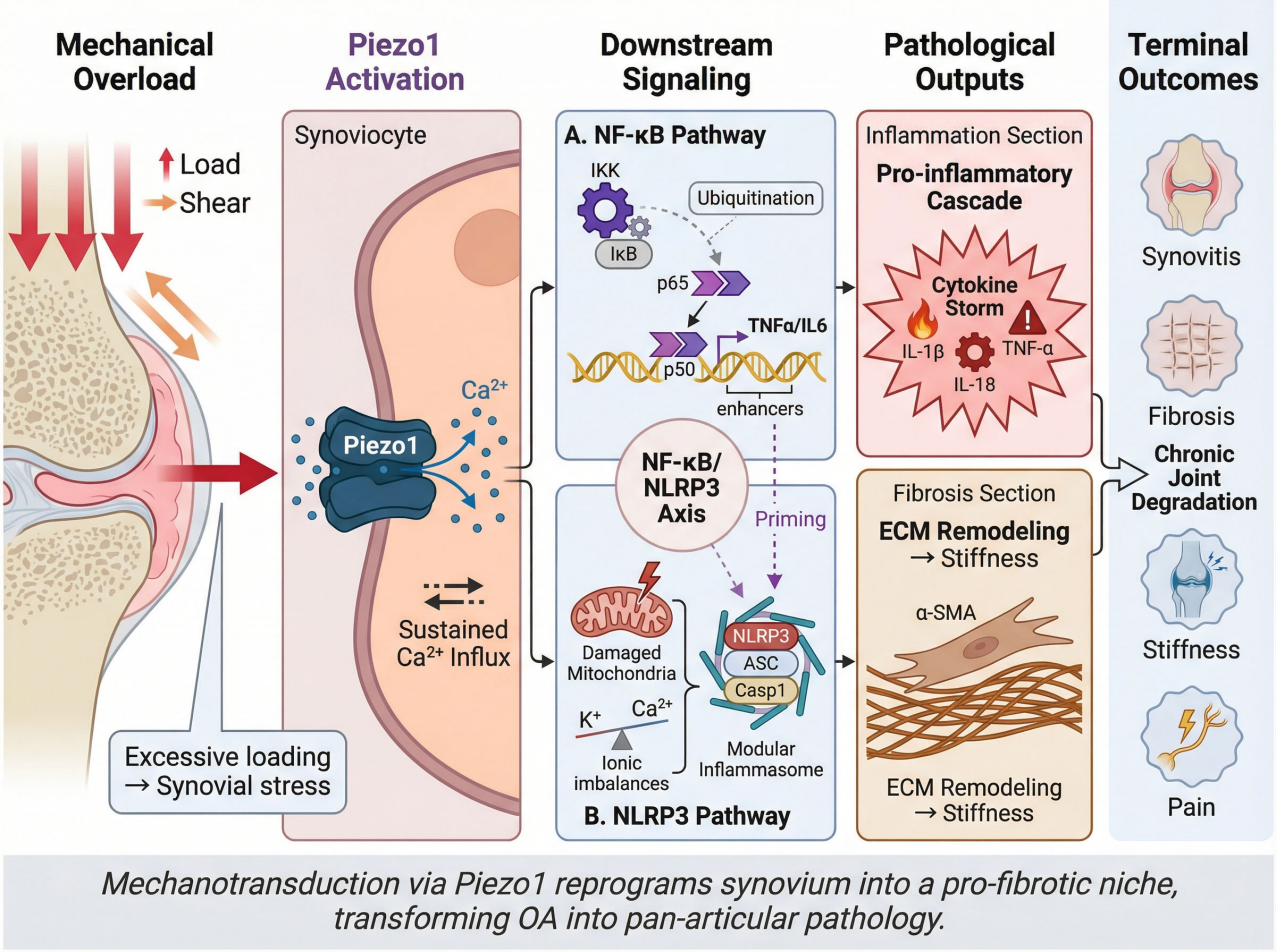
Mechanical overload–induced Piezo1–NF-κB/NLRP3 axis drives synovitis and fibrosis in osteoarthritis.

Piezo1 is a mechanically activated ion channel that responds rapidly to membrane tension and mechanical deformation. Under conditions of excessive mechanical loading, Piezo1 activation leads to sustained calcium influx in synovial cells, initiating downstream inflammatory signaling. Accumulating evidence indicates that Piezo1 functions as a critical upstream trigger of NF-κB activation in the synovium, thereby promoting transcription of pro-inflammatory mediators (52). Notably, Piezo1-driven NF-κB signaling is tightly coupled to activation of the NLRP3 inflammasome. NF-κB primes inflammasome components, while mechanical stress–induced ionic fluxes and mitochondrial perturbations provide the second signal required for inflammasome assembly (53). The resulting NF-κB/NLRP3 axis amplifies inflammatory cytokine release and fosters a profibrotic microenvironment within the synovium (54). This process contributes to synovitis, extracellular matrix deposition, and fibrotic remodeling, which exacerbate joint stiffness and pain. The involvement of Piezo1-mediated NF-κB signaling highlights a critical conceptual shift in OA pathogenesis: mechanical overload does not merely damage cartilage but actively reprograms synovial tissue toward chronic inflammation and fibrosis. Through this mechanism, OA extends beyond a cartilage-centric disorder and emerges as a disease of the entire joint organ (**Figure 1**).

#### 3.1.2 TRPV4 and primary cilia: structural gating of NF-κB/COL2 signaling

**Figure 2:**
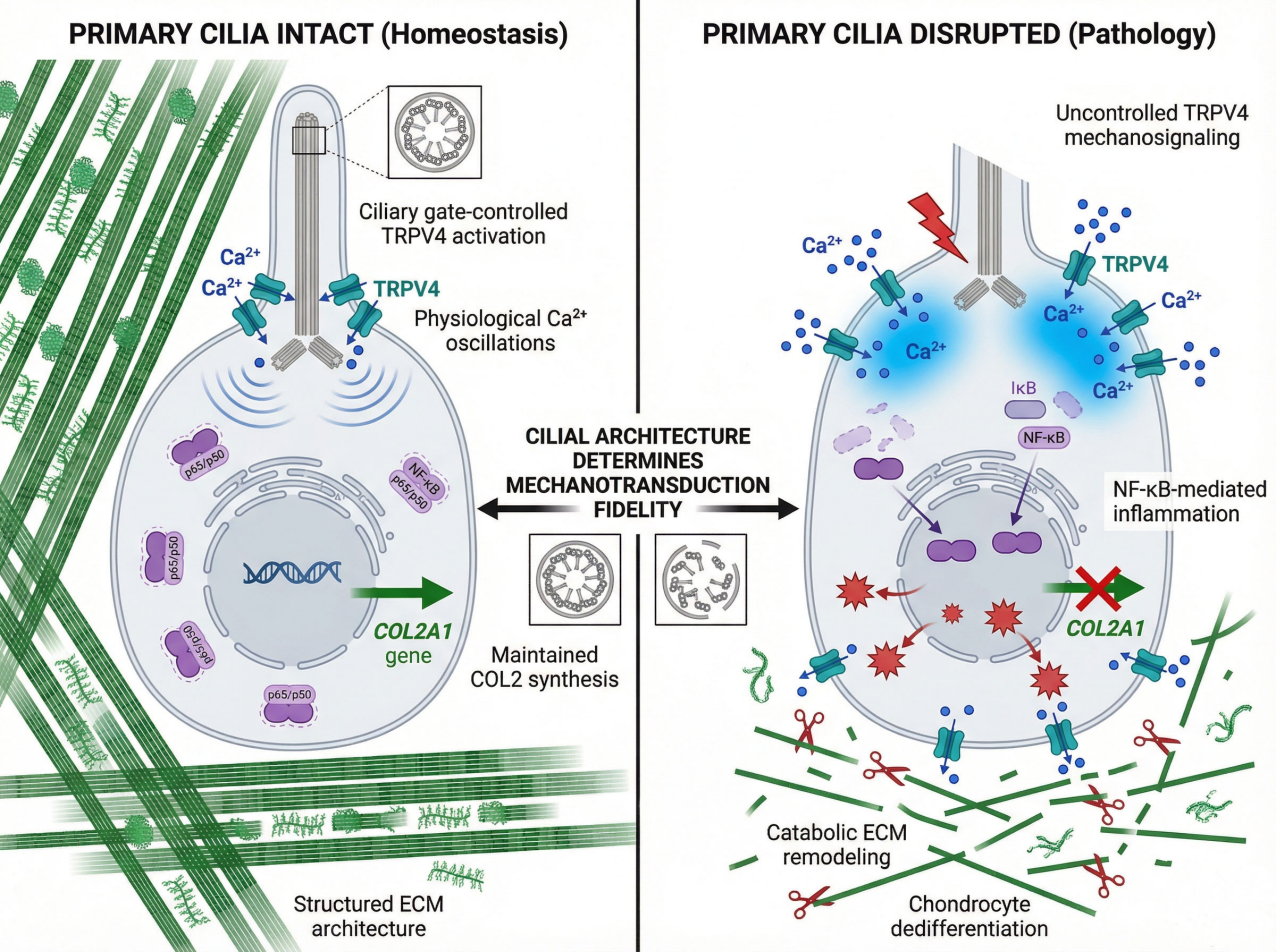
Primary cilia structurally gate TRPV4-mediated mechanotransduction in chondrocytes. TRPV4 functions as a mechanosensitive ion channel whose signaling output is tightly regulated by the integrity of primary cilia. When primary cilia are intact, TRPV4-mediated calcium influx is finely tuned, supporting extracellular matrix homeostasis by maintaining type II collagen (COL2) expression and restraining NF-κB activation. In contrast, disruption of primary cilia abolishes this structural gating, leading to aberrant TRPV4 signaling, excessive calcium influx, and enhanced NF-κB–dependent inflammatory transcription accompanied by loss of anabolic gene expression. This cilia-dependent modulation highlights how cellular architecture determines whether mechanical stimuli preserve cartilage integrity or promote inflammatory degeneration in osteoarthritis.

In contrast to Piezo1, which predominantly mediates pathological responses to mechanical overload, transient receptor potential vanilloid 4 (TRPV4) participates in more nuanced mechanosensitive signaling in chondrocytes. TRPV4 activation is closely regulated by the integrity of primary cilia—specialized, microtubule-based organelles that function as biomechanical and biochemical signaling hubs. Primary cilia act as structural gatekeepers that constrain and fine-tune TRPV4-mediated calcium signaling. When ciliary architecture is intact, TRPV4 activation supports cartilage homeostasis by maintaining type II collagen (COL2) expression and extracellular matrix integrity. However, disruption of primary cilia alters TRPV4 signal output, skewing downstream signaling toward NF-κB–dependent inflammatory transcription and loss of anabolic gene expression (55). This cilia-dependent modulation of TRPV4 activity underscores the importance of cellular architecture in shaping mechanotransduction outcomes. Rather than serving as a simple on–off switch, TRPV4 signaling operates within a structural context that determines whether mechanical stimuli preserve ECM homeostasis or promote inflammatory degeneration via NF-κB activation (**Figure 2**).

### 3.2 Moderate mechanical stress as a protective input: suppression of ferroptosis via NF-κB p65/GPX4 signaling

**Figure 3:**
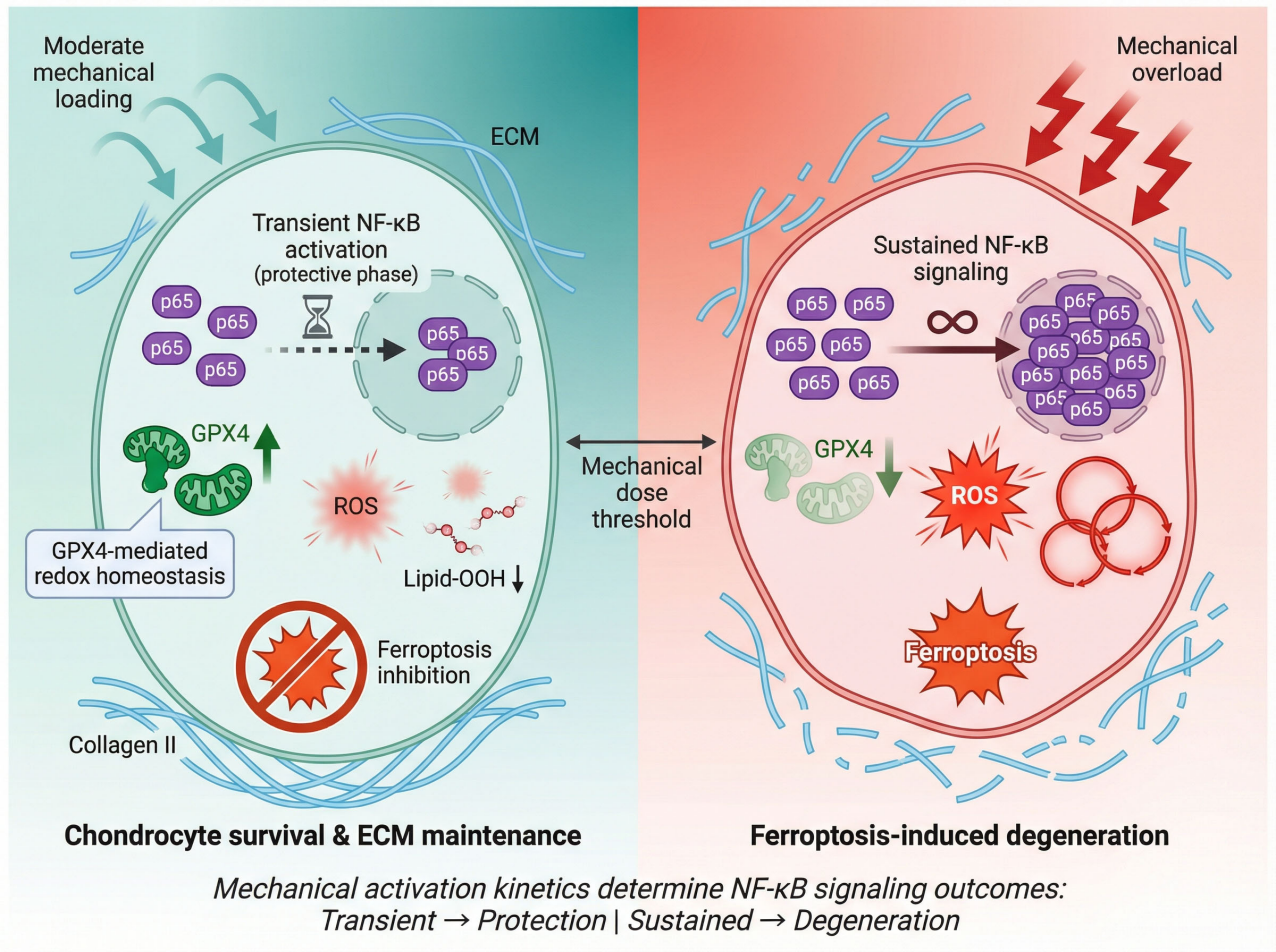
Moderate mechanical stress suppresses chondrocyte ferroptosis via the NF-κB p65/GPX4 axis. Moderate mechanical loading elicits a transient and low-amplitude activation of NF-κB signaling in chondrocytes, which engages adaptive transcriptional programs rather than chronic inflammatory responses. Under this condition, NF-κB p65 promotes antioxidant defense by sustaining GPX4 expression, thereby limiting lipid peroxidation and suppressing ferroptotic cell death. In contrast, mechanical overload induces sustained NF-κB activation, leading to oxidative imbalance, GPX4 depletion, enhanced lipid peroxidation, and ferroptosis-associated cartilage degeneration. This dose- and kinetics-dependent signaling paradigm highlights NF-κB as a context-sensitive integrator of mechanical cues that bifurcates chondrocyte fate toward either stress resilience and cartilage homeostasis or degenerative cell death in osteoarthritis.

While excessive mechanical stress drives inflammatory degeneration, emerging evidence indicates that moderate mechanical loading can exert protective effects on articular cartilage. This paradox highlights the importance of mechanical dose and temporal patterning in determining NF-κB signaling outcomes. Under moderate mechanical stress, transient activation of NF-κB appears to engage adaptive transcriptional programs rather than chronic inflammatory responses. One notable mechanism involves regulation of ferroptosis, a form of iron-dependent lipid peroxidation–driven cell death increasingly implicated in OA pathogenesis. Moderate mechanical loading has been shown to suppress chondrocyte ferroptosis by modulating the NF-κB p65/GPX4 axis, thereby preserving antioxidant defenses and cellular viability. This protective role of NF-κB challenges the traditional view of NF-κB as an exclusively deleterious mediator in OA. Instead, it suggests that NF-κB functions as a context-sensitive signal integrator whose downstream effects depend on activation kinetics (**Figure 3**). Short-lived or low-amplitude NF-κB activation may support redox balance and stress resilience, whereas prolonged activation under mechanical overload promotes inflammation, inflammasome signaling, and tissue degeneration.

### 3.3 Mechanical input shapes NF-κB activation curves and pathological outcomes

Collectively, these findings support a dose-dependent model in which mechanical stress sculpts NF-κB activation curves to determine joint fate. Mechanical overload induces sustained and high-amplitude NF-κB activation, driving inflammatory transcription, NLRP3 inflammasome priming, synovial fibrosis, and progressive cartilage degeneration. In contrast, moderate mechanical stress elicits transient and reversible NF-κB signaling that favors antioxidant defenses, suppression of ferroptosis, and maintenance of tissue homeostasis. This conceptual framework reconciles seemingly contradictory observations regarding mechanical loading in OA and emphasizes the importance of temporal and quantitative dimensions of mechanotransduction (56). From a translational perspective, it also suggests that therapeutic strategies should aim not at indiscriminate suppression of mechanical signaling or NF-κB activity, but at restoring physiological activation patterns that preserve joint integrity. A schematic illustration depicting the relationship between mechanical stress intensity, NF-κB activation dynamics, and downstream biological outcomes would help visualize this dose–time–effect paradigm.

## 4 Innate immunity as a convergent route to NF-κB activation in osteoarthritis

Innate immune signaling constitutes an important convergence point through which diverse stress signals may be translated into sustained NF-κB activation in osteoarthritis signaling. Unlike acute inflammatory responses to infection, innate immune activation in OA is predominantly sterile and driven by endogenous danger signals generated by mechanical overload, mitochondrial dysfunction, and cellular injury. These signals engage pattern recognition receptors and cytosolic sensors that amplify inflammatory transcriptional programs and reinforce joint degeneration (35, 57). Among these pathways, the cGAS–STING axis and Toll-like receptor (TLR) signaling represent two principal routes linking cellular stress to NF-κB–mediated pathology. **Table 1** summarizes representative studies on the upstream mechanisms linking mechanical stress and innate immune activation to NF-κB signaling in osteoarthritis. The table highlights the cited references, experimental models, and the principal NF-κB-related findings across these studies.

**Table 1:** Representative studies on upstream mechanical and innate immune inputs converging on NF-κB signaling in osteoarthritis.

Section/theme	Representative reference	PMID	*In vitro* model	*In vivo* model	NF-κB-related finding	Relevance to review
Piezo1 and mechanoinflammation	Lee et al., 2021 (10), *PNAS*	33758095	Articular chondrocytes	OA model reported in study	Inflammatory signaling sensitizes Piezo1-dependent mechanotransduction in OA	Supports the idea that mechanical stress and inflammatory signaling are biologically coupled
Piezo1–NF-κB/NLRP3 in synovitis/fibrosis	Yu et al., 2025 (54), *Cell Signal*	40221068	Mechanically stressed synovial cell system	OA synovitis/fibrosis model reported in study	Mechanical overload promotes NF-κB/NLRP3-mediated synovitis and fibrosis through Piezo1	Connects excessive mechanical loading to sterile inflammation
TRPV4/primary cilia structural gating	Sun et al., 2025 (55), *Cell Signal*	40975506	Chondrocyte mechanotransduction system	OA pathogenesis model reported in study	Primary cilia integrity governs TRPV4-mediated NF-κB/COL2 signaling	Shows structural gating of inflammatory vs homeostatic mechanotransduction
Piezo1/TRPV4 ion-channel crosstalk	Steinecker-Frohnwieser et al., 2023 (56), *Int J Mol Sci*	37175575	Primary human healthy and OA chondrocytes	OA model reported in study	Piezo1/TRPV4 communicate with each other and may be altered in OA	Human *in vitro* support for upstream mechanosensitive signaling
TRPM2–Ca²^+^–cGAS–STING–NF-κB loop	Sun et al., 2025 (36), *J Adv Res*	39505144	OA chondrocyte damage model	OA model reported in study	TRPM2-mediated calcium dysregulation activates cGAS–STING–NF-κB	Strong bridge between stress, innate sensing, and NF-κB
STING in experimental OA	Shin et al., 2023 (59), *Arthritis Res Ther*	37259103	Not emphasized in title/abstract snippet	Mouse experimental OA model	STING mediates experimental OA and mechanical allodynia	Gives *in vivo* support for innate immune amplification
HMGB1/TLR4/NF-κB axis	Li et al., 2024 (64), *J Biochem Mol Toxicol*	37943572	OA inflammatory cell/chondrocyte model	OA animal model reported in study	Forsythoside B alleviates OA through HMGB1/TLR4/NF-κB and Keap1/Nrf2/HO-1	Illustrates DAMP-driven sterile inflammation converging on NF-κB
TLR2/NF-κB signaling	Yang et al., 2022 (58), *Front Pharmacol*	36034791	Cell-based OA inflammatory model	OA animal model reported in study	Celastrol ameliorates OA via regulating TLR2/NF-κB signaling	Supports extracellular danger signaling as an upstream NF-κB input
TLR2/NF-κB with explicit dual-model design	Liu et al., 2017 (147), Oncotarget	28418842	*In vitro* OA-related cell model	*In vivo* OA model	TLR2/NF-κB contributes to degenerative knee OA	Useful because it explicitly includes both *in vitro* and *in vivo* arms

### 4.1 Calcium–mitochondria–DNA sensing: the cGAS–STING–NF-κB feed-forward loop

#### 4.1.1 TRPM2-mediated Ca²^+^ influx links cellular stress to cGAS–STING and NF-κB

Transient receptor potential melastatin 2 (TRPM2) is a calcium-permeable ion channel that responds to oxidative stress and inflammatory cues. In osteoarthritic chondrocytes, TRPM2 activation induces sustained intracellular Ca²^+^ influx, disrupting calcium homeostasis and imposing mitochondrial stress. Elevated mitochondrial calcium levels compromise mitochondrial integrity, leading to mitochondrial membrane depolarization, excessive reactive oxygen species generation, and the release of mitochondrial DNA (mtDNA) into the cytosol. Cytosolic mtDNA serves as a potent danger signal that activates cyclic GMP–AMP synthase (cGAS), triggering downstream stimulation of stimulator of interferon genes (STING) (58). While STING activation classically induces type I interferon responses, accumulating evidence indicates that STING also robustly activates NF-κB signaling in the context of sterile inflammation. In OA, this TRPM2–Ca²^+^–mitochondria–cGAS–STING cascade culminates in NF-κB–dependent transcription of inflammatory mediators, matrix-degrading enzymes, and stress response genes (36). Importantly, this pathway forms a feed-forward amplification loop. NF-κB activation exacerbates oxidative stress, inflammatory signaling, and cellular injury, thereby further promoting TRPM2 activation, mitochondrial dysfunction, and mtDNA release. Through this self-reinforcing circuit, transient stress signals are converted into persistent inflammatory programs that drive progressive cartilage degeneration. This feed-forward loop provides a mechanistic framework linking ion channel–mediated calcium dysregulation to innate immune sensing and chronic NF-κB activation in OA.

#### 4.1.2 STING/NF-κB coupling as an inflammatory amplifier in OA

**Figure 4:**
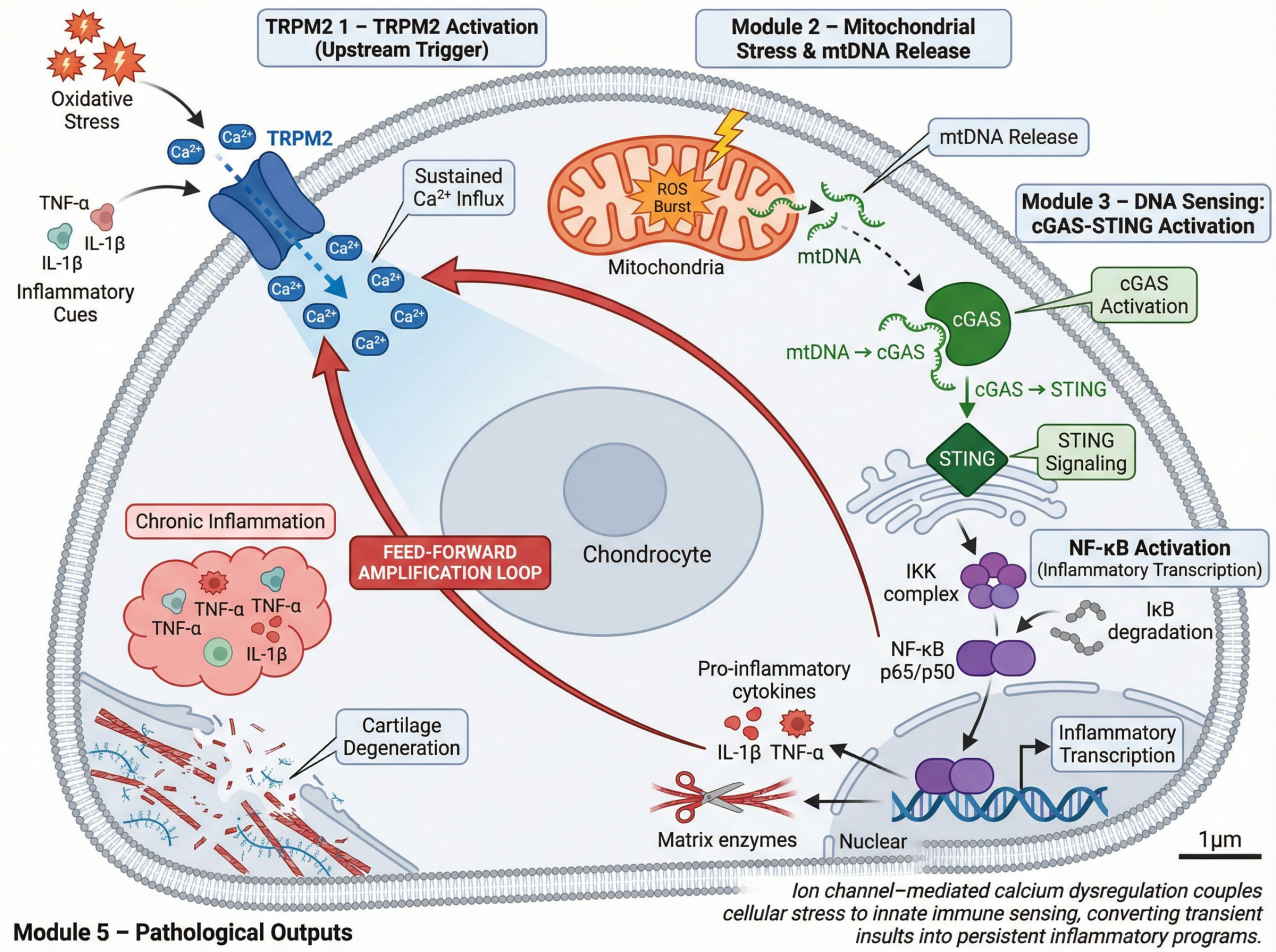
TRPM2-mediated Ca²^+^ influx activates a cGAS–STING–NF-κB feed-forward loop in osteoarthritis. TRPM2 functions as a stress-responsive calcium channel that links oxidative and inflammatory stimuli to innate immune activation in osteoarthritic chondrocytes. Sustained TRPM2-mediated Ca²^+^ influx disrupts calcium homeostasis and induces mitochondrial stress, leading to mitochondrial membrane depolarization, excessive reactive oxygen species generation, and cytosolic release of mitochondrial DNA (mtDNA). Cytosolic mtDNA activates the cGAS–STING pathway, which, in addition to interferon signaling, robustly engages NF-κB–dependent inflammatory transcription. NF-κB activation further exacerbates oxidative stress, mitochondrial dysfunction, and cellular injury, thereby reinforcing TRPM2 activation and mtDNA release. This self-amplifying feed-forward loop converts transient cellular stress into persistent inflammatory signaling, driving chronic cartilage degeneration in osteoarthritis.

Beyond its canonical role in antiviral immunity, STING has emerged as an important mediator of sterile inflammation in degenerative diseases. In OA, STING signaling operates at the intersection of DNA sensing, mitochondrial stress, and inflammatory transcription (42). While STING activation can induce interferon-stimulated genes, its coupling to NF-κB is particularly relevant for chronic joint inflammation (59). STING-dependent NF-κB activation amplifies the expression of pro-inflammatory cytokines, chemokines, and catabolic enzymes, thereby intensifying tissue damage (60). This dual signaling capacity positions STING as an inflammatory amplifier that bridges innate immune detection of cellular damage with NF-κB–driven degenerative responses. From a conceptual standpoint, STING–NF-κB coupling highlights how OA co-opts antiviral defense machinery to sustain sterile inflammation, transforming intracellular stress signals into extracellular inflammatory cascades that propagate joint pathology (**Figure 4**).

### 4.2 DAMP–TLR–NF-κB axis: HMGB1/TLR4 and TLR2-driven inflammation

**Figure 5:**
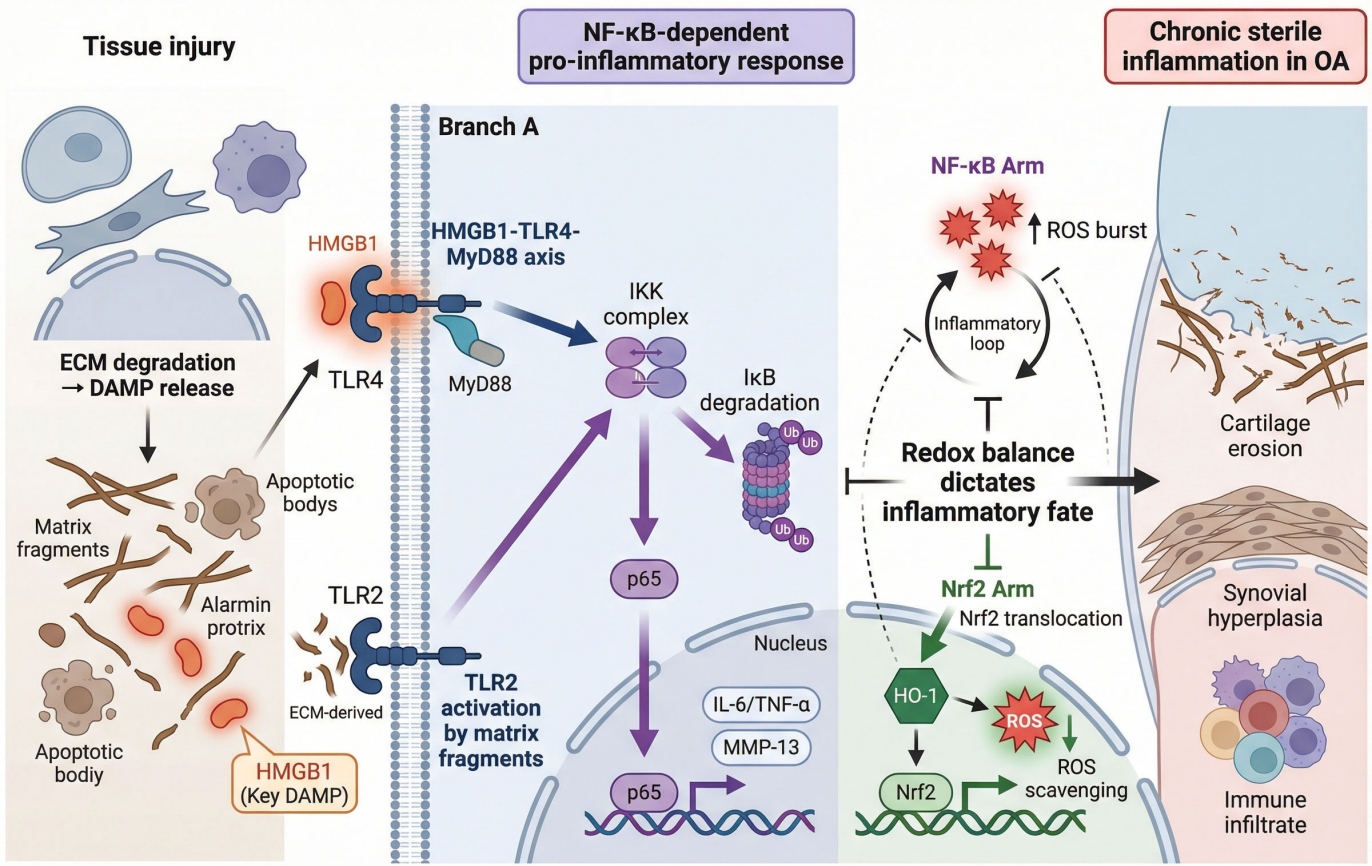
DAMP–TLR–NF-κB signaling sustains sterile inflammation in osteoarthritis. Extracellular danger-associated molecular patterns (DAMPs) released from injured cartilage, degraded extracellular matrix, and dying cells activate innate immune signaling in osteoarthritis. High mobility group box 1 (HMGB1), a prototypical DAMP, binds to TLR4 and triggers MyD88-dependent signaling cascades that converge on NF-κB activation, driving inflammatory gene expression and matrix degradation. In parallel, endogenous ligands and matrix fragments activate TLR2, further reinforcing NF-κB–dependent inflammatory responses in joint tissues. The pathological outcome of DAMP-induced signaling is critically modulated by redox balance, as Nrf2/HO-1–mediated antioxidant responses counteract NF-κB–driven inflammation. Disruption of this balance favors persistent sterile inflammation, contributing to cartilage degeneration and synovial pathology in osteoarthritis.

In addition to cytosolic DNA sensing, extracellular danger-associated molecular patterns (DAMPs) represent a major source of innate immune activation in OA. Tissue injury, ECM degradation, and cell death release endogenous ligands that engage Toll-like receptors on chondrocytes, synovial fibroblasts, and immune cells, leading to NF-κB activation. High mobility group box 1 (HMGB1) is a prototypical DAMP abundantly released during cartilage damage and synovial inflammation (61). Binding of HMGB1 to TLR4 activates MyD88-dependent signaling cascades that converge on NF-κB, driving inflammatory gene expression and matrix degradation (62, 63). Importantly, HMGB1/TLR4/NF-κB signaling is tightly linked to oxidative stress, and its pathological effects are modulated by the antioxidant Nrf2/HO-1 axis (64). Crosstalk between Nrf2 and NF-κB determines whether DAMP-induced signaling results in adaptive resolution or persistent inflammation, underscoring the importance of redox balance in OA progression. TLR2 represents another critical innate immune receptor implicated in OA. Activation of TLR2 by endogenous ligands or cartilage-derived matrix fragments promotes NF-κB–dependent inflammatory responses in joint tissues (45, 65). Notably, several natural polysaccharides and bioactive compounds have been shown to attenuate OA progression by suppressing TLR2/NF-κB signaling, providing pharmacological evidence for the pathological relevance of this axis (**Figure 5**). Together, HMGB1/TLR4 and TLR2 signaling exemplify how extracellular damage signals converge on NF-κB to sustain sterile inflammation within the osteoarthritic joint.

### 4.3 Cytokine and immune ligand inputs to NF-κB in the OA microenvironment

**Figure 6:**
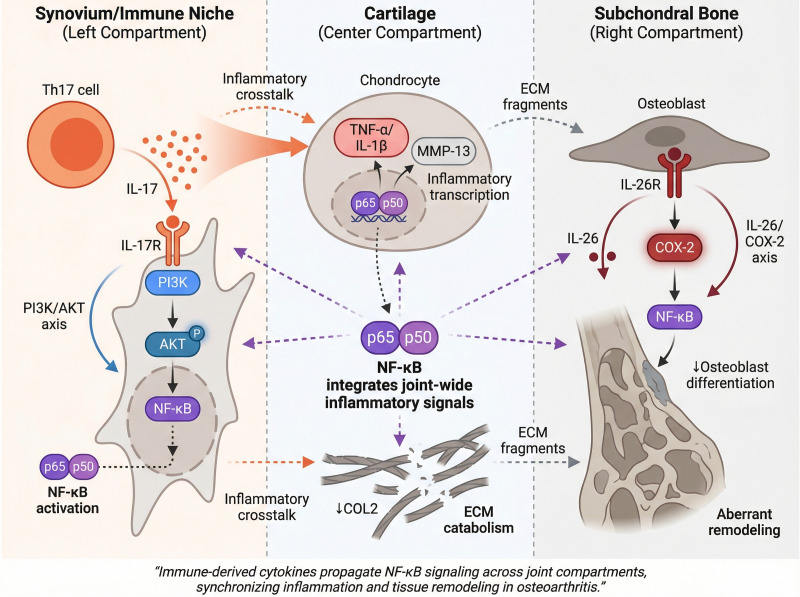
Cytokine and immune ligand inputs amplify NF-κB signaling across joint compartments in osteoarthritis. The osteoarthritic joint microenvironment is enriched with immune-derived cytokines that reinforce NF-κB activation and coordinate multicompartmental pathology. IL-17 acts as a key pro-inflammatory cytokine linking immune activation to cartilage degeneration by engaging PI3K/AKT signaling, which cooperates with NF-κB to amplify inflammatory transcription and catabolic processes in chondrocytes. In parallel, IL-26 activates COX2 and NF-κB signaling in osteoblasts, suppressing osteoblast differentiation and contributing to dysregulated subchondral bone remodeling. Together, these cytokine-driven pathways extend NF-κB signaling beyond cartilage and synovium to involve subchondral bone, reinforcing osteoarthritis as a whole-joint inflammatory disease.

The OA joint microenvironment is enriched with cytokines and immune ligands that further reinforce NF-κB activation and shape disease progression. Among these, IL-17 has emerged as a key pro-inflammatory cytokine linking immune activation to cartilage degeneration. IL-17 signaling engages the PI3K/AKT pathway, which cooperates with NF-κB to amplify inflammatory transcription and catabolic processes (31, 66). This convergence provides a mechanistic rationale for combination therapeutic strategies targeting both IL-17 signaling and NF-κB–associated pathways (67). Interleukin-26 (IL-26) represents a distinct immune ligand with implications for bone metabolism and joint remodeling. IL-26 has been shown to activate COX2 and NF-κB signaling in osteoblasts, thereby attenuating osteoblast differentiation and contributing to dysregulated bone remodeling in OA (68). This pathway highlights how immune-derived cytokines extend NF-κB signaling beyond cartilage and synovium to influence subchondral bone biology, reinforcing the concept of OA as a multicompartmental inflammatory disease (**Figure 6**).

### 4.4 The metabolic–immunological interface: immunometabolic reprogramming, mitochondrial dynamics, and NAD^+^-dependent regulation of NF-κB in osteoarthritis

NF-κB signaling in osteoarthritis is shaped not only by mechanical stress and innate immune ligands, but also by the metabolic configuration of the responding cell. In this sense, OA should be viewed as a disorder in which inflammatory signaling and metabolic adaptation are tightly interwoven rather than separable processes (69, 70). Chondrocytes, synovial fibroblasts, macrophages, and subchondral bone-associated cells all undergo state-dependent metabolic reprogramming under chronic stress conditions, including altered glucose utilization, redox imbalance, lipid remodeling, and mitochondrial dysfunction. These metabolic changes do not merely accompany inflammation; they influence the amplitude, persistence, and downstream consequences of NF-κB activation (71–74). Thus, the OA joint microenvironment represents an immunometabolic niche in which inflammatory and metabolic signals are mutually reinforcing. This framework is particularly relevant to macrophage-rich synovial inflammation and obesity-associated OA. Immune activation is closely linked to metabolic state, and macrophage polarization is increasingly recognized as an immunometabolic process rather than a purely cytokine-defined phenotype (75, 76). Pro-inflammatory macrophage programs are frequently associated with glycolytic bias, altered mitochondrial function, and sustained NF-κB activity, whereas inflammation-resolving states depend on a different bioenergetic and redox configuration. Similar principles may also apply to stressed chondrocytes and fibroblast-like synoviocytes, where metabolic adaptation influences inflammatory sensitivity and matrix-catabolic behavior. In obesity-related OA, systemic low-grade inflammation, altered lipid handling, and metabolic stress likely amplify NF-κB-dependent signaling across multiple joint compartments, providing a mechanistic bridge between systemic metabolism and local tissue degeneration.

Mitochondria are central determinants of this metabolic–inflammatory interface. Importantly, their role extends beyond generic “mitochondrial dysfunction.” Mitochondrial fusion–fission balance, mitophagy efficiency, calcium buffering capacity, reactive oxygen species production, and mitochondrial DNA retention all shape the cellular threshold for NF-κB activation and signal persistence (77–79). When mitochondrial quality control is preserved, stress-induced NF-κB activation may remain transient and adaptive. By contrast, excessive fission, defective mitophagy, persistent ROS generation, and mtDNA leakage promote prolonged inflammatory signaling through redox amplification and DNA-sensing pathways such as cGAS–STING. In this context, mitochondrial dynamics function not merely as downstream consequences of OA-related stress, but as upstream signaling determinants that bias NF-κB output toward either resolution or chronic inflammatory entrenchment.

An additional metabolic layer of NF-κB regulation involves NAD^+^ homeostasis (80, 81). NAD^+^ is a central metabolic cofactor linking energy metabolism, redox control, DNA damage responses, and stress adaptation (82, 83). In aged or chronically stressed tissues, NAD^+^ depletion may impair homeostatic mechanisms that normally restrain inflammatory signaling. One important connection is the Sirt1/NF-κB axis, in which Sirt1-mediated deacetylation suppresses NF-κB transcriptional activity. Reduced NAD^+^ availability may therefore weaken sirtuin-dependent feedback control and favor persistent inflammatory transcription (84–86). In parallel, chronic DNA damage and PARP activation may further consume NAD^+^, reinforcing metabolic stress and inflammatory persistence. Although NAD^+^-centered regulation has not yet been fully defined in OA, this axis provides a plausible mechanistic bridge linking aging, mitochondrial stress, and sustained NF-κB activation.

Taken together, these observations support a broader view in which NF-κB is embedded within an immunometabolic signaling network rather than functioning as an isolated inflammatory effector. Future studies integrating metabolic flux, mitochondrial state, redox biology, and cell-type-resolved inflammatory profiling will be essential for defining how metabolic context determines NF-κB-driven disease trajectories in osteoarthritis.

## 5 NF-κB-driven cartilage degeneration: ECM breakdown and chondrocyte fate decisions

Articular cartilage degeneration represents a central pathological feature of osteoarthritis, and NF-κB signaling plays an important role in mediating this processsignaling. Beyond its well-established function in inflammatory gene expression, NF-κB acts as a master regulator that coordinates extracellular matrix (ECM) remodeling and governs chondrocyte fate decisions (87). These two processes—matrix breakdown and cellular fate reprogramming—are tightly interlinked and jointly determine the irreversibility of cartilage damage in OA (88). Representative studies on extracellular matrix remodeling and chondrocyte fate regulation are summarized in **Table 2**. The table highlights the cited references, experimental models, and major NF-κB-related findings relevant to cartilage degeneration in osteoarthritis.

**Table 2:** Representative studies linking NF-κB to extracellular matrix degradation and chondrocyte fate decisions in osteoarthritis.

Section/theme	Representative reference	PMID	*In vitro* model	*In vivo* model	NF-κB-related finding
ECM degradation/inflammatory catabolism	Miao et al., 2024 (141), *J Orthop Translat*	38867741	Chondrocytes	Not fully specified in snippet	SPARCL1 promotes ECM degradation and inflammation via TNF/NF-κB
STING-driven senescence/apoptosis/ECM loss	Guo et al., 2021 (24), *Cell Death Dis*	33414452	OA chondrocytes	OA model reported in study	STING promotes senescence, apoptosis, and ECM degradation via NF-κB
Chondrocyte senescence	Jie et al., 2025 (38), *Int J Mol Med*	41104872	Chondrocytes	OA murine model reported in article/PMC text	PINK1 overexpression suppresses p38 MAPK/NF-κB and attenuates senescence
Chondrocyte apoptosis	Liang et al., 2025 (112), Oral Dis	40013619	TMJ chondrocyte-related system	Joint-specific OA model	DDX5 modulates TNF-induced NF-κB signaling and apoptosis
Ferroptosis and OA protection	Zhao et al., 2023 (25), *J Orthop Translat*	37188001	OA-related cell model	OA model reported in study	FOXO3 attenuates OA by suppressing ferroptosis through NF-κB/MAPK inactivation
Mechanical stress/ferroptosis/NF-κB p65–GPX4 axis	Han et al., 2024 (39), Sci Rep	38429394	OA-related cell model	OA model reported in study	Moderate mechanical stress suppresses ferroptosis via NF-κB p65/GPX4 signaling
PI3K/AKT/NF-κB and chondrocyte dysfunction	Deng et al., 2024 (31), Biomed Pharmacothe	38262147	Chondrocytes	OA model reported in study	Sakuranetin reduces inflammation and dysfunction via PI3K/AKT/NF-κB

### 5.1 Collapse of ECM homeostasis downstream of NF-κB signaling

Under physiological conditions, articular cartilage maintains a finely balanced equilibrium between anabolic synthesis and catabolic turnover of ECM components, including type II collagen and aggrecan. NF-κB signaling contributes to this balance by enabling adaptive responses to transient stress (89). However, in OA, persistent NF-κB activation induces a transcriptional reprogramming that favors catabolic dominance (90). Rather than acting through isolated downstream effectors, NF-κB drives a coordinated degradation program by simultaneously upregulating matrix-degrading enzymes, inflammatory mediators, and stress response genes (91). This transcriptional shift suppresses anabolic signaling while amplifying ECM breakdown, ultimately destabilizing cartilage architecture. Importantly, NF-κB–mediated ECM degradation is not merely a consequence of inflammation but actively feeds back to sustain NF-κB activation, as matrix fragments and damage-associated signals further stimulate innate immune and inflammatory pathways (92). Through this mechanism, NF-κB establishes a self-perpetuating loop that locks cartilage into a degenerative trajectory.

### 5.2 Chondrocyte senescence: coupling p38 MAPK/NF-κB signaling with mitochondrial quality control

**Figure 7:**
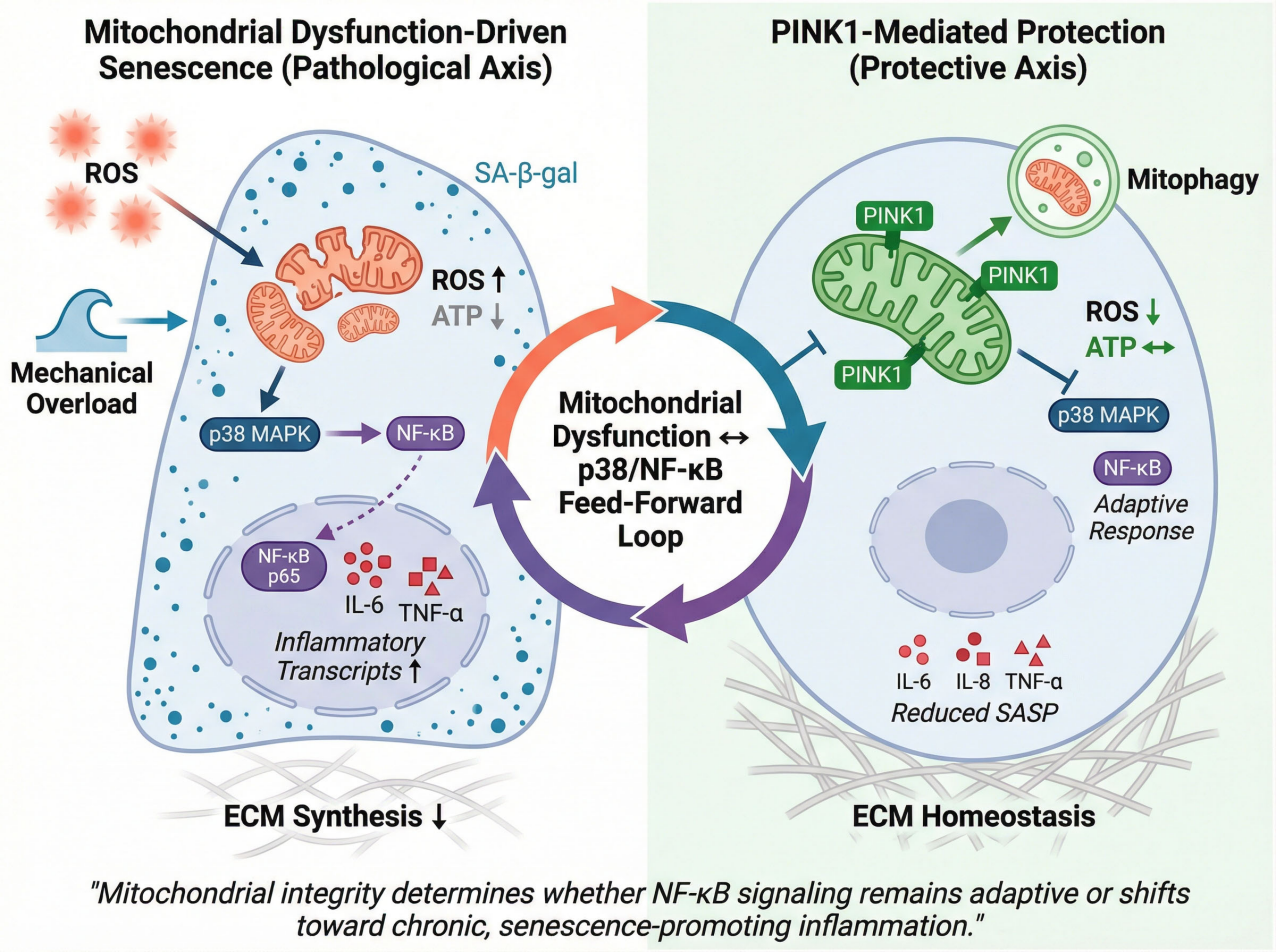
Mitochondrial quality control modulates p38 MAPK/NF-κB–driven chondrocyte senescence in osteoarthritis. Chondrocyte senescence is a key pathological feature of osteoarthritis and is driven by sustained inflammatory signaling and mitochondrial dysfunction. Oxidative stress and mechanical overload impair mitochondrial quality control, leading to excessive reactive oxygen species production and bioenergetic failure. Mitochondrial stress activates the p38 MAPK/NF-κB axis, promoting senescence-associated secretory phenotype (SASP) expression and reinforcing inflammatory signaling. In contrast, PINK1-mediated mitochondrial surveillance preserves mitochondrial integrity, suppresses p38 MAPK and NF-κB activation, and attenuates chondrocyte senescence. These findings highlight a functional coupling between mitochondrial quality control and inflammatory transcription, positioning mitochondrial integrity as a critical determinant of whether NF-κB signaling drives adaptive stress responses or irreversible cellular aging in osteoarthritis.

Cellular senescence has emerged as a key contributor to OA progression, characterized by irreversible growth arrest, metabolic rewiring, and acquisition of a senescence-associated secretory phenotype (SASP). NF-κB is a central driver of the SASP program, linking intracellular stress to sustained inflammatory output (93). In chondrocytes, activation of the p38 MAPK/NF-κB axis represents a critical pathway promoting senescence under conditions of oxidative stress and mechanical overload (94). Mitochondrial dysfunction plays a pivotal role in this process. Impaired mitochondrial quality control leads to excessive reactive oxygen species production and bioenergetic failure, which in turn reinforces p38 MAPK and NF-κB signaling (95, 96). PINK1-mediated mitochondrial surveillance provides an important counter-regulatory mechanism in this context. Overexpression of PINK1 has been shown to attenuate p38 MAPK/NF-κB activation, thereby alleviating chondrocyte senescence (38). This finding highlights a functional coupling between mitochondrial quality control and inflammatory transcriptional regulation. When mitochondrial homeostasis is preserved, NF-κB activation remains constrained and adaptive; when mitochondrial stress accumulates, NF-κB signaling shifts toward a chronic, senescence-promoting mode. Thus, mitochondrial integrity acts as a critical determinant of whether NF-κB signaling drives reversible stress responses or irreversible cellular aging (**Figure 7**).

### 5.3 Aging and cellular senescence as contextual modifiers of NF-κB signaling in osteoarthritis

Aging is the strongest risk factor for osteoarthritis, yet its role in NF-κB-centered disease mechanisms extends beyond simple chronological exposure to mechanical wear (97, 98). Rather than acting merely as a background condition, aging reshapes the signaling landscape of joint tissues and serves as a critical contextual modifier of NF-κB responsiveness, persistence, and downstream output. In aged cartilage, synovium, and subchondral bone, chronic low-grade sterile inflammation, impaired stress adaptation, mitochondrial dysfunction, and progressive cellular senescence collectively create a permissive environment for sustained NF-κB activation (99, 100). From this perspective, age-related OA should be understood not only as a mechanically accumulated disorder, but also as a disease of inflammaging-driven signal amplification.

The concept of inflammaging is particularly relevant to this framework. Aging tissues are characterized by a persistent, low-grade inflammatory tone even in the absence of overt infection, driven by cumulative cellular damage, DAMP release, altered immunometabolism, and declining homeostatic resilience (57, 101). In the osteoarthritic joint, this inflammatory background may lower the activation threshold of NF-κB signaling and prolong its response to mechanical stress, oxidative injury, and innate immune ligands. As a result, stimuli that might otherwise elicit transient adaptive signaling in younger tissues may instead trigger exaggerated or persistent inflammatory programs in aged joint cells. In this way, inflammaging provides an important explanation for why age modifies both the magnitude and the pathogenic consequences of NF-κB activation in OA.

Another important but often underappreciated mechanism linking aging to NF-κB signaling is telomere dysfunction. Telomere attrition and telomere-associated DNA damage are hallmarks of cellular aging and have been implicated in chondrocyte dysfunction and senescence (102, 103). Dysfunctional telomeres trigger a persistent DNA damage response, which can reinforce growth arrest, pro-inflammatory transcriptional reprogramming, and senescence-associated phenotypes. Although telomere biology has not been fully integrated into NF-κB-centered models of OA, accumulating evidence suggests that genotoxic stress and DNA damage signaling can intersect with inflammatory pathways, thereby helping couple age-related replicative stress to persistent NF-κB activation (24, 104, 105). This connection may be particularly relevant in long-lived, stress-exposed joint cells, where telomere dysfunction acts less as an isolated aging marker than as a mechanism that biases tissues toward chronic inflammatory signaling and impaired repair.

Cellular senescence further amplifies this process through the senescence-associated secretory phenotype (SASP) (106, 107). NF-κB is a major regulator of SASP-associated transcription, controlling the expression of pro-inflammatory cytokines, chemokines, proteases, and matrix-remodeling factors (108, 109). Once established, the SASP does not merely reflect a passive endpoint of cellular aging; rather, it acts as an active paracrine driver of tissue degeneration. SASP factors released by senescent chondrocytes or stromal cells can reinforce NF-κB activation in neighboring chondrocytes, synovial fibroblasts, and immune cells, thereby propagating inflammatory signaling, matrix catabolism, and secondary senescence across the joint microenvironment. This creates a self-reinforcing feed-forward loop in which NF-κB promotes senescence-associated secretion, and SASP-associated mediators further sustain NF-κB activity. Such a loop provides a mechanistic basis for persistent low-grade inflammation, multicompartmental degeneration, and progressive loss of tissue homeostasis in aged osteoarthritic joints.

Importantly, senescence is unlikely to be uniform across the joint or even within a single cell population. Different subsets of chondrocytes and synovial cells may exhibit variable senescence burden, DNA damage accumulation, metabolic reserve, and inflammatory priming, suggesting that aging-related NF-κB dysregulation is also likely to be heterogeneous at the single-cell level. This heterogeneity may partly explain why identical mechanical or inflammatory stimuli yield divergent outcomes in different cells and why age-related OA is marked by substantial intercellular variability in catabolic behavior, survival capacity, and stress vulnerability. Taken together, these observations support the view that aging and cellular senescence are not peripheral modifiers, but central determinants of how NF-κB signaling is initiated, sustained, and decoded in osteoarthritis.

### 5.4 Chondrocyte apoptosis: inflammatory cues and regulatory proteins

**Figure 8:**
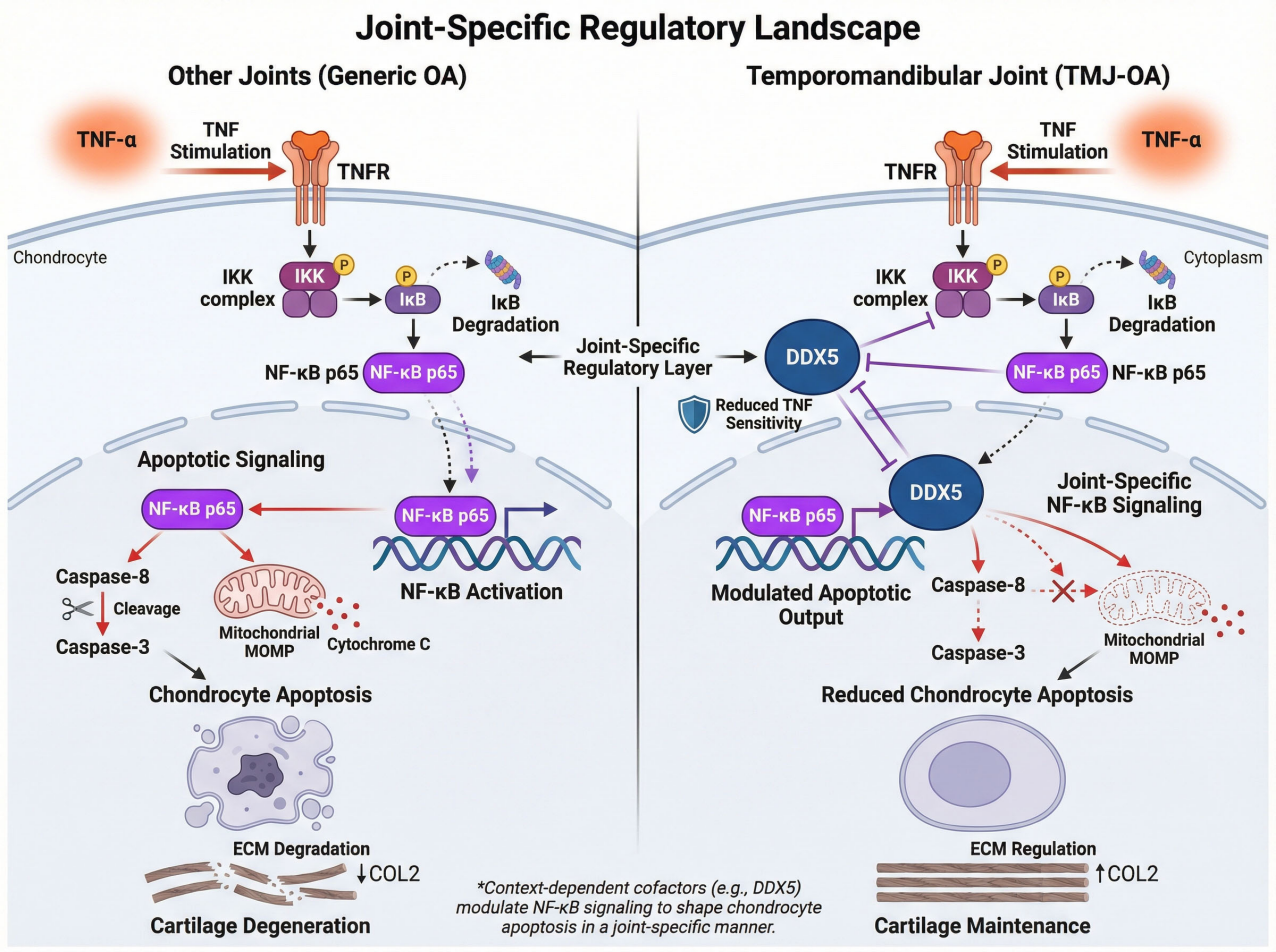
DDX5 modulates TNF-induced NF-κB signaling to regulate chondrocyte apoptosis in temporomandibular joint osteoarthritis. Inflammatory cytokines such as tumor necrosis factor (TNF) activate NF-κB signaling pathways that intersect with apoptotic machinery to influence chondrocyte survival in osteoarthritis. In the temporomandibular joint, the RNA helicase DDX5 functions as a context-specific regulator of TNF-induced NF-κB signaling, thereby modulating chondrocyte susceptibility to apoptosis and extracellular matrix degradation. This joint-specific regulatory layer highlights how NF-κB–driven apoptotic responses are shaped by local signaling environments and regulatory cofactors. Temporomandibular joint osteoarthritis thus provides a unique model illustrating that distinct joint types exhibit differential NF-κB regulatory landscapes, which in turn determine cartilage cell fate and disease progression.

In addition to senescence, NF-κB signaling also influences chondrocyte apoptosis, particularly under conditions of intense or unresolved inflammatory stress (110). Pro-inflammatory cytokines such as tumor necrosis factor (TNF) activate NF-κB signaling pathways that intersect with apoptotic machinery, shaping cell survival outcomes (111). The RNA helicase DDX5 has been identified as a regulatory factor modulating TNF-induced NF-κB signaling in temporomandibular joint osteoarthritis (TMJ-OA). In this joint-specific context, DDX5-mediated regulation of NF-κB activity influences chondrocyte susceptibility to apoptosis and ECM degradation (112). This example underscores two important concepts: first, that NF-κB-driven apoptotic regulation is modulated by context-specific cofactors; and second, that different joint types may exhibit distinct NF-κB regulatory landscapes. TMJ-OA thus provides a valuable model illustrating how joint-specific signaling environments shape NF-κB–dependent cartilage pathology (**Figure 8**).

### 5.5 Ferroptosis intersects with NF-κB signaling in cartilage degeneration

**Figure 9:**
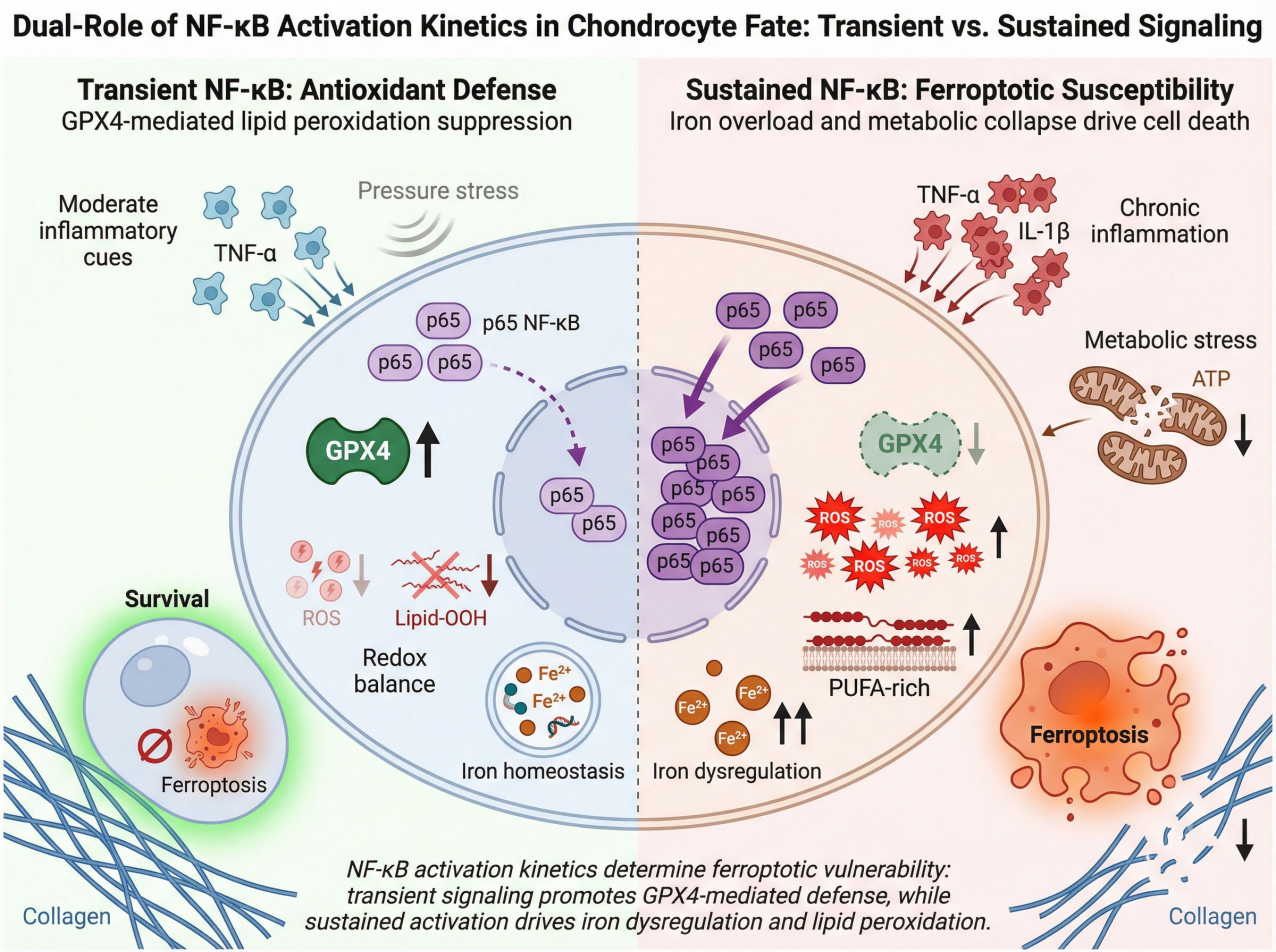
NF-κB intersects with ferroptotic signaling to regulate cartilage degeneration in osteoarthritis. Ferroptosis is an iron-dependent form of regulated cell death driven by lipid peroxidation and has emerged as a contributor to osteoarthritis-associated cartilage degeneration. NF-κB signaling intersects with ferroptotic pathways by integrating inflammatory, oxidative, and metabolic cues in chondrocytes. A key point of convergence is the NF-κB p65/GPX4 axis, in which GPX4 functions as a central antioxidant enzyme that suppresses lipid peroxidation and ferroptosis. Transient or moderate NF-κB activation sustains GPX4-mediated redox defense and protects chondrocytes from ferroptotic damage. In contrast, sustained NF-κB signaling under chronic inflammatory or metabolic stress promotes oxidative imbalance, iron dysregulation, and lipid peroxidation, tipping the balance toward ferroptotic cell death and cartilage degeneration. These interactions position NF-κB as a central node linking inflammation to ferroptosis in osteoarthritis.

Ferroptosis, an iron-dependent form of regulated cell death driven by lipid peroxidation, has recently been implicated in OA-associated cartilage degeneration (113). NF-κB signaling intersects with ferroptotic pathways at multiple levels, integrating inflammatory, oxidative, and metabolic signals to influence chondrocyte survival (114). A key point of convergence is the NF-κB p65/GPX4 axis (39). GPX4 functions as a central antioxidant enzyme that suppresses lipid peroxidation and ferroptosis (115). NF-κB can modulate GPX4 expression and activity, thereby indirectly controlling ferroptotic vulnerability. Under conditions of moderate or transient NF-κB activation, upregulation of antioxidant defenses may protect chondrocytes from ferroptotic damage. In contrast, sustained NF-κB signaling under inflammatory or metabolic stress promotes oxidative imbalance, iron dysregulation, and lipid peroxidation, tipping the balance toward ferroptotic cell death. Moreover, NF-κB-driven metabolic reprogramming further sensitizes chondrocytes to ferroptosis by altering redox homeostasis and lipid metabolism (116). These interactions position NF-κB as a central node that links inflammation to emerging forms of regulated cell death in OA (**Figure 9**).

### 5.6 NF-κB defines chondrocyte fate trajectories in osteoarthritis

Taken together, these findings support a model in which NF-κB functions as a context-dependent mediator of chondrocyte fate regulation. The same NF-κB signaling machinery can drive divergent outcomes—ECM degradation, senescence, apoptosis, or ferroptosis—depending on the context of activation. Several factors appear to dictate these fate trajectories. First, the source of upstream stimuli is critical: mechanical overload, innate immune activation, and metabolic inflammation engage distinct NF-κB-associated signaling modules. Second, the intensity and duration of NF-κB activation determine whether responses remain adaptive or become pathological. Third, the intrinsic cellular state, including age, metabolic background, and mitochondrial health, modulates NF-κB responsiveness and downstream signaling bias (117). This context-dependent signaling logic explains why indiscriminate inhibition of NF-κB may yield mixed therapeutic outcomes. Instead, selectively reshaping NF-κB activation patterns to favor protective over degenerative programs may represent a more effective strategy for preserving cartilage integrity. Understanding how NF-κB integrates diverse stress signals to direct chondrocyte fate decisions therefore provides a conceptual framework for the development of precision therapies in osteoarthritis.

### 5.7 Divergent chondrocyte fate decisions under shared NF-κB inputs: a cell-state-dependent model

Although senescence, apoptosis, and ferroptosis are all discussed as NF-κB-associated outcomes in osteoarthritis, these fates should not be interpreted as parallel deterministic consequences of a uniform signal (25, 39). A central unresolved question in OA biology is why apparently similar upstream stimuli—such as mechanical overload, inflammatory cytokines, oxidative stress, or innate immune activation—can drive distinct cellular outcomes in different chondrocytes. A more plausible model is that NF-κB functions as a context-sensitive signaling interpreter whose biological output is filtered through cell-state-specific determinants rather than dictated by stimulus identity alone (118, 119).

One major determinant of fate selection is mitochondrial fitness. Chondrocytes with preserved mitochondrial quality control may tolerate transient NF-κB activation and maintain adaptive stress responses, whereas cells with impaired mitophagy, persistent mitochondrial depolarization, or excessive reactive oxygen species generation are more likely to enter senescence or apoptosis (46, 120). In this setting, mitochondrial dysfunction not only amplifies NF-κB signaling but also biases downstream transcriptional programs toward irreversible cellular damage. This concept is consistent with observations linking mitochondrial stress, p38 MAPK/NF-κB activation, and senescence-associated phenotypes in OA cartilage. Thus, mitochondrial status may act as a key gatekeeper that determines whether NF-κB signaling remains adaptive or becomes degenerative.

A second determinant is redox buffering capacity. NF-κB outputs are strongly influenced by the balance between oxidative burden and antioxidant defense. Chondrocytes with sufficient Nrf2 activity, glutathione availability, and GPX4-dependent lipid peroxide detoxification may remain resistant to lethal oxidative damage even when inflammatory signaling is activated (121, 122). By contrast, cells with compromised redox buffering are more susceptible to oxidative amplification and ferroptotic execution. In this framework, ferroptosis should not be viewed simply as another downstream event triggered by NF-κB, but as the result of NF-κB signaling acting within a permissive metabolic and redox context. The NF-κB p65/GPX4 axis therefore represents not only a mechanistic linkage between inflammation and ferroptosis, but also an example of how fate choice depends on cellular reserve capacity (39). Iron handling and lipid metabolic configuration provide an additional layer of divergence (25, 123). Ferroptotic susceptibility is critically shaped by intracellular iron availability, lipid composition, and membrane peroxidation vulnerability. Chondrocytes with dysregulated iron metabolism, impaired ferritin buffering, or elevated polyunsaturated lipid content may be predisposed to ferroptotic collapse under inflammatory stress, whereas other cells exposed to similar upstream signals may instead undergo senescence-associated remodeling or apoptotic death. This helps explain why shared inflammatory or mechanical inputs do not produce uniform outcomes across the cartilage cell population.

Epigenetic and transcriptional state also likely influence how NF-κB signaling is decoded. The same NF-κB dimer does not necessarily engage identical target genes in all cells, because transcriptional output depends on chromatin accessibility, cofactor availability, enhancer usage, and pre-existing inflammatory priming (124, 125). Aging, prior cytokine exposure, and senescence-associated chromatin remodeling may all alter the transcriptional landscape through which NF-κB operates, thereby biasing cell fate decisions. In this sense, NF-κB signaling in OA should be viewed as state dependent not only at the level of upstream stimulus exposure, but also at the level of target gene accessibility and transcriptional competence. These considerations support a cell-state-dependent model in which shared NF-κB inputs are translated into divergent outputs according to mitochondrial integrity, redox reserve, iron and lipid metabolism, and epigenetic configuration. At the single-cell level, osteoarthritic chondrocytes are therefore unlikely to respond uniformly even when exposed to the same microenvironmental stressor (73, 126, 127). Instead, pre-existing heterogeneity in stress adaptation capacity and metabolic vulnerability likely determines whether NF-κB signaling culminates in matrix-degrading persistence, senescence, apoptosis, or ferroptosis. This framework has important translational implications. Rather than attempting to suppress NF-κB indiscriminately, future therapeutic strategies may need to identify and target the specific cellular states in which NF-κB signaling becomes pathogenic. Integrating single-cell multi-omics, spatial transcriptomics, and functional stress mapping will be particularly important for resolving these fate biases and for defining clinically relevant NF-κB-dependent OA subtypes.

## 6 NF-κB in immune microenvironment remodeling: macrophage polarization and chemokine axes

The osteoarthritic joint is increasingly recognized as an immunologically active microenvironment rather than an immune-privileged site. Innate immune cells—particularly macrophages—accumulate in synovial tissue and subchondral bone, where they interact dynamically with chondrocytes, fibroblasts, and osteoblast-lineage cells. NF-κB signaling plays a central role in orchestrating this immune remodeling by regulating macrophage polarization states and chemokine-driven cellular crosstalk (128, 129). Through these mechanisms, NF-κB extends its influence beyond cell-intrinsic effects to shape joint-wide inflammatory circuits.

### 6.1 Macrophage polarization as a bidirectional regulator of osteoarthritis

Macrophages exhibit remarkable plasticity, adopting pro-inflammatory (M1-like) or anti-inflammatory and tissue-repairing (M2-like) phenotypes in response to microenvironmental cues. In OA, an imbalance favoring M1 polarization contributes to sustained inflammation, cartilage degradation, and pain. NF-κB signaling is a dominant determinant of macrophage activation status, integrating signals from DAMPs, cytokines, and mechanical stress (121, 130). Triggering receptor expressed on myeloid cells 2 (TREM2) has emerged as a critical modulator of macrophage polarization in OA. TREM2 signaling promotes a shift from M1 to M2 phenotypes, thereby dampening inflammatory responses and supporting tissue repair. Mechanistically, TREM2-mediated macrophage reprogramming involves suppression of NF-κB activity and downstream regulation of chemokine expression, particularly the NF-κB/CXCL3 axis (71). By restraining CXCL3-driven inflammatory amplification, TREM2 limits immune cell recruitment and inflammatory signaling within the joint. This bidirectional regulation underscores the dual nature of NF-κB in macrophages. While excessive NF-κB activation drives pathogenic inflammation, controlled modulation of NF-κB signaling—such as through TREM2—can tilt macrophage polarization toward a protective, inflammation-resolving state. These findings position macrophage NF-κB signaling not merely as a driver of OA progression but also as a potential lever for immunomodulatory intervention.

### 6.2 Obesity-related OA: CDK5–PPARγ/NF-κB axis and macrophage–chondrocyte crosstalk

Metabolic inflammation represents an important modifier of OA pathogenesis, particularly in the context of obesity. Obesity-associated OA is characterized by systemic low-grade inflammation that exacerbates local joint pathology through altered immune cell behavior and chondrocyte stress responses. NF-κB signaling serves as a key molecular link between metabolic cues and inflammatory joint degeneration. Cyclin-dependent kinase 5 (CDK5) has been identified as an upstream regulator of the PPARγ/NF-κB axis in obesity-related OA. CDK5-mediated modulation of PPARγ activity disrupts anti-inflammatory transcriptional programs and favors NF-κB–driven inflammatory signaling (37). This shift promotes pro-inflammatory macrophage polarization while simultaneously sensitizing chondrocytes to apoptotic stimuli. The resulting macrophage–chondrocyte crosstalk amplifies joint degeneration: macrophage-derived inflammatory mediators activate NF-κB in chondrocytes, accelerating apoptosis and ECM breakdown, while damaged cartilage releases additional DAMPs that further stimulate macrophage NF-κB signaling. This feed-forward loop illustrates how metabolic inflammation transforms NF-κB from a localized inflammatory mediator into a systemic amplifier of OA progression. Importantly, it highlights obesity-related OA as a distinct disease subtype in which immunometabolic regulation of NF-κB plays a disproportionately large role.

### 6.3 Chemokine modules downstream of NF-κB: linking synovium, cartilage, and subchondral bone

Chemokines represent critical downstream effectors through which NF-κB signaling coordinates multicompartmental interactions within the joint (131). By regulating chemokine expression, NF-κB shapes immune cell recruitment, spatial organization, and inter-tissue communication (132, 133). CXCL3 is a prominent NF-κB–regulated chemokine implicated in macrophage behavior and synovial inflammation. As discussed above, NF-κB/CXCL3 signaling influences macrophage polarization and inflammatory amplification within the synovium. Dysregulated CXCL3 expression sustains immune cell infiltration and reinforces local NF-κB activation, contributing to chronic synovitis. CXCL2 represents another NF-κB–dependent chemokine with distinct pathological implications. In the subchondral bone compartment, NF-κB–driven CXCL2 expression promotes osteoclast recruitment and activation, thereby enhancing bone resorption and remodeling (134, 135). These alterations in subchondral bone structure modify joint biomechanics, indirectly increasing mechanical stress on articular cartilage and accelerating degeneration. Together, the CXCL2 and CXCL3 axes illustrate how NF-κB–regulated chemokine modules function as connective threads linking the synovium, cartilage, and subchondral bone. Rather than acting in isolation, these compartments engage in reciprocal signaling loops mediated by chemokines, immune cells, and mechanical feedback. Through this integrated network, NF-κB orchestrates joint-wide immune remodeling that sustains OA progression.

## 7 Therapeutic targeting: modulating the NF-κB hub to restore joint homeostasis

Given the central role of NF-κB as an integrative signaling hub in osteoarthritis, therapeutic strategies aimed at modulating this pathway have attracted increasing attention. However, direct inhibition of NF-κB has yielded limited clinical success, largely due to its pleiotropic roles in tissue homeostasis and immune defense. Emerging evidence instead supports a network-based intervention paradigm, in which upstream triggers, NF-κB core signaling, and coupled regulatory pathways are selectively targeted to reshape pathological signaling toward joint homeostasis. To provide a coherent framework for these approaches, we categorize current and emerging interventions according to their hierarchical position relative to the NF-κB hub.

### 7.1 Targeting upstream triggers of NF-κB activation

One therapeutic strategy focuses on attenuating the upstream signals that initiate or sustain NF-κB activation in OA. Mechanical stress–sensing mechanisms and innate immune receptors represent two major classes of such triggers. Mechanosensitive ion channels, including Piezo1, TRPV4, and TRPM2, act as primary transducers of mechanical and oxidative stress into intracellular signaling cascades (136). While direct pharmacological modulation of these channels in OA remains largely exploratory, their identification as upstream NF-κB activators highlights new intervention opportunities. Rather than complete blockade, fine-tuning channel activity to restore physiological mechanotransduction may help normalize NF-κB activation patterns without compromising essential cellular functions. Innate immune receptors provide another entry point for upstream intervention. Suppression of TLR2/TLR4 signaling or neutralization of their endogenous ligands, such as HMGB1, has been shown to attenuate NF-κB–driven inflammation in experimental OA models (137, 138). Similarly, targeting the cGAS–STING pathway offers a means to interrupt DNA sensing–mediated NF-κB activation arising from mitochondrial stress and cellular damage (139). Collectively, these approaches aim to reduce the inflammatory load converging on NF-κB, thereby dampening downstream degenerative cascades.

### 7.2 Targeting the NF-κB core and its coupled regulatory pathways

The majority of therapeutic evidence in OA converges on pathways that regulate NF-κB activity indirectly through interconnected signaling networks. These strategies seek to rebalance NF-κB signaling rather than abolish it.

#### 7.2.1 Sirtuin–NF-κB axis

Sirtuins, particularly Sirt1, function as critical modulators of inflammatory transcription by deacetylating NF-κB subunits and transcriptional cofactors. Activation of the Sirt1/NF-κB axis has emerged as a promising strategy for restoring joint homeostasis (140, 141). Notably, pulsed electromagnetic field (PEMF) therapy has been shown to ameliorate OA progression through activation of Sirt1 and subsequent suppression of NF-κB signaling. This example is particularly compelling because it integrates a physical intervention with a defined molecular mechanism, underscoring the translational potential of targeting NF-κB regulatory nodes beyond conventional pharmacology.

#### 7.2.2 Nrf2/HO-1–NF-κB axis

The Nrf2/HO-1 pathway represents a major antioxidant and cytoprotective system that counterbalances NF-κB–driven inflammation. Several pharmacological agents and natural compounds exert therapeutic effects in OA by simultaneously activating Nrf2 signaling and suppressing NF-κB activity. AM1241 exemplifies this dual-pathway modulation, engaging the Nrf2/HO-1 axis while inhibiting NF-κB–mediated inflammatory responses (142). In addition, multiple natural products reported in OA models converge on this antioxidant–anti-inflammatory coupling (143). Rather than acting as isolated anti-inflammatory agents, these compounds restore redox balance and dampen inflammatory amplification, highlighting the importance of oxidative stress control in NF-κB–centered therapeutic strategies.

#### 7.2.3 PI3K/AKT–NF-κB and mTOR coupling

The PI3K/AKT pathway plays a pivotal role in modulating NF-κB activation, cell survival, and metabolic reprogramming in OA. Several small molecules and bioactive compounds have been shown to alleviate OA pathology by suppressing PI3K/AKT–dependent NF-κB signaling. Sakuranetin and pulchinenoside C reduce inflammation and cartilage degeneration by inhibiting the PI3K/AKT/NF-κB axis (144, 145). CN7:1h further extends this regulatory network by modulating both NF-κB and mTOR signaling, linking inflammatory control to metabolic regulation and autophagic processes (146, 147). Fostamatinib exemplifies a broader network-targeting approach, simultaneously influencing MAPK/NF-κB and AKT/mTOR pathways to preserve cartilage homeostasis (148, 149). Importantly, combination strategies provide additional insight into pathway interdependence. The synergistic effects observed with parecoxib and ilomastat highlight how IL-17–driven PI3K/AKT/NF-κB signaling can be more effectively suppressed through coordinated targeting of upstream cytokine signaling and downstream inflammatory execution (67, 150). Such findings support the rationale for rational combination therapies designed around NF-κB network topology.

### 7.3 Natural products and ethnopharmacology: evidence quality and mechanistic convergence

Natural products and ethnopharmacological agents constitute a substantial proportion of experimental OA therapeutics. Although structurally diverse, many of these compounds exhibit a striking mechanistic convergence: they ultimately suppress NF-κB signaling and its coupled pathways, including PI3K/AKT, Nrf2/HO-1, STING, and TLR-mediated signaling. Rather than viewing these agents as heterogeneous or nonspecific, their consistent convergence on NF-κB–associated nodes suggests that they act as network modulators capable of restoring signaling balance. Importantly, the strength of evidence supporting these interventions varies considerably. Most studies remain at the cellular or animal model level, while only a limited number incorporate human samples or clinical observations (151). A graded evaluation of evidence—from *in vitro* mechanistic studies to *in vivo* validation and clinical relevance—highlights both the promise and limitations of natural product–based therapies. Integrating human-derived data, such as cytokine profiling or patient tissue analyses, will be essential for advancing these agents beyond preclinical proof-of-concept.

### 7.4 Controversies, limitations, and unresolved issues in interpreting NF-κB signaling in osteoarthritis

Despite the central role of NF-κB in osteoarthritis pathogenesis, several important controversies and limitations complicate its interpretation as a therapeutic target. First, NF-κB signaling should not be regarded as uniformly deleterious. Although sustained activation clearly promotes inflammatory amplification, matrix catabolism, fibrosis, and cell death, transient or low-level NF-κB activity may also support adaptive stress responses, survival signaling, and antioxidant defense under selected conditions (18, 152). This duality is particularly relevant in mechanically loaded tissues, where complete pathway suppression could disrupt physiological stress adaptation rather than restore homeostasis. Accordingly, the therapeutic objective may be better framed as normalization of pathological NF-κB persistence rather than indiscriminate pathway blockade.

Second, the translational history of NF-κB inhibition highlights a major gap between mechanistic plausibility and clinical success. Broad NF-κB suppression has not emerged as a successful disease-modifying strategy in OA or related chronic degenerative conditions, in part because NF-κB regulates essential homeostatic and immune functions in addition to pathological inflammation. Furthermore, OA is highly heterogeneous, and not all patients are likely to exhibit a dominant NF-κB-driven disease program (18, 152, 153). Timing may also be critical, as anti-inflammatory intervention at structurally advanced stages may be insufficient to reverse established joint damage. In addition, many agents described as NF-κB inhibitors act indirectly through upstream kinases, redox pathways, or pleiotropic stress-response mechanisms, making it difficult to attribute observed effects specifically to NF-κB blockade. These issues underscore the need for pharmacodynamic biomarkers, mechanism-based patient stratification, and clearer target-engagement evidence in future translational studies.

Third, the contribution of non-canonical NF-κB signaling in OA remains incompletely resolved. Compared with the canonical pathway, which is strongly supported across chondrocytes, synovium, and inflammatory microenvironments, evidence for non-canonical signaling is still limited, compartment-specific, and in some cases indirect (18, 154, 155). Although non-canonical NF-κB may participate in chronic remodeling, immune differentiation, or bone-related processes, its causal contribution to the broader OA disease trajectory remains uncertain. For this reason, conclusions regarding non-canonical pathway involvement should remain cautious until more rigorous cell-type-specific and functional studies become available.

Finally, the field is constrained by several methodological limitations. Many studies document NF-κB activation in parallel with OA progression, but fewer establish whether it functions as an initiating driver, a signal amplifier, or a secondary consequence of tissue injury. Common experimental models, such as IL-1β-stimulated chondrocytes or surgically induced OA in rodents, reproduce selected inflammatory or biomechanical features but do not fully capture the chronic, heterogeneous, and multicompartmental nature of human disease (156, 157). Bulk tissue analyses may further obscure cellular heterogeneity, while pharmacological studies are often limited by insufficient specificity. Importantly, suppression of p65 phosphorylation or inflammatory marker expression does not necessarily equate to meaningful disease modification. Addressing these issues will require more rigorous integration of genetic perturbation, cell-type-resolved analysis, spatial profiling, and clinically relevant outcome measures.

Taken together, these considerations suggest that NF-κB remains a highly informative mechanistic axis in OA, but one whose interpretation requires greater attention to duality, evidence hierarchy, pharmacological specificity, and disease heterogeneity. **Table 3** summarizes representative studies addressing immune microenvironment remodeling and NF-κB-targeted therapeutic interventions in osteoarthritis. It provides an overview of the cited references, experimental models, mechanistic findings, and translational implications across these studies.

**Table 3:** Representative studies on immune microenvironment remodeling and NF-κB-targeted therapeutic strategies in osteoarthritis.

Section/theme	Representative reference	PMID	*In vitro* model	*In vivo* model	NF-κB-related finding	Translational significance
Obesity-related OA/macrophage–chondrocyte crosstalk	Li et al., 2025 (37), *Cell Mol Biol Lett*	41233759	Macrophage–chondrocyte related assays	Obesity-related OA model reported in article summary	CDK5 regulates PPARγ/NF-κB, promotes M1 polarization and chondrocyte apoptosis	Strong paper for immunometabolic amplification around NF-κB
Macrophage polarization and anti-inflammatory remodeling	Shen et al., 2023 (145), *Int Immunopharmacol*	37182454	Chondrocytes and macrophages	Mice model of OA	Suramin acts on Nrf2/HO-1 and NF-κB and promotes M2 polarization	Connects immune remodeling to therapeutic NF-κB modulation
Sirt1/NF-κB targeting by physical therapy	Zhou et al., 2025 (140), *Arthritis Res Ther*	39953605	Not fully specified in snippet	OA mouse model	PEMF ameliorates OA via the Sirt1/NF-κB pathway	Example that NF-κB modulation is not only pharmacologic
Nrf2/HO-1–NF-κB dual targeting	Zou et al., 2025 (142), *Mol Med*	39794700	Chondrocytes	Mice OA model	AM1241 inhibits inflammation and ECM degradation through Nrf2/HO-1 and NF-κB	Good example of pathway-coupled intervention with cell and animal support
NF-κB inhibition by natural product	Lin et al., 2019 (143), *Front Pharmacol*	31214026	IL-1β-induced chondrocytes	Mouse OA model	Nobiletin suppresses NF-κB signaling and attenuates OA	Classic natural-product example
PI3K/AKT/NF-κB targeting	Hu et al., 2025 (144), *J Cell Mol Med*	40746264	Cell-based OA model	OA model reported in article	Pulchinenoside C attenuates OA by inhibiting PI3K/AKT/NF-κB	Useful for pathway-network intervention table
NF-κB/mTOR coupling	Wang et al., 2025 (146), *J Cell Mol Med*	39875323	OA-related cell model	OA model reported in article	CN7:1h inhibits NF-κB and mTOR, promotes autophagy, reduces apoptosis and ECM degradation	Supports the network-based intervention argument
MAPK/NF-κB and AKT/mTOR dual targeting	Zhang et al., 2025 (148), *Int Immunopharmacol*	39756165	TMJ cell-based OA model	TMJ-OA model	Fostamatinib maintains cartilage homeostasis through MAPK/NF-κB and AKT/mTOR	Example of broader network modulation rather than isolated NF-κB blockade

## 8 Clinical implications and future directions

Recognizing NF-κB as an important integrative signaling mediator in osteoarthritis carries important clinical implications. Rather than representing a uniform disease entity, OA encompasses multiple mechanistically distinct subtypes in which NF-κB signaling is engaged through different upstream drivers and operates within diverse cellular contexts. Understanding these differences is essential for translating mechanistic insights into effective, personalized therapeutic strategies.

### 8.1 Patient stratification and biomarkers: toward identifying NF-κB-associated OA patterns

A major translational challenge in osteoarthritis is the absence of robust stratification frameworks that link molecular mechanisms to clinical phenotypes. Although the NF-κB-oriented signaling framework outlined in this review may offer a useful conceptual lens for thinking about disease heterogeneity, it should not yet be interpreted as a validated basis for defining discrete clinical OA subtypes (158, 159). At present, most available evidence remains preclinical or mechanistically oriented, and there is still limited support from longitudinal human cohorts, standardized biomarker studies, or interventional trials for NF-κB-based patient classification. Nevertheless, existing studies suggest that NF-κB-related signaling may be differentially enriched across several clinically relevant contexts. For example, OA associated with joint malalignment, post-traumatic injury, or chronic biomechanical overload may show greater engagement of mechanotransduction-linked inflammatory pathways, including ion channel–associated signaling (160). Likewise, OA with prominent synovitis or inflammatory features may involve stronger activation of innate immune pathways, including TLR- and cGAS–STING-related signaling (121, 161, 162). In obesity-associated or metabolically dysregulated OA, NF-κB activation may be shaped by systemic low-grade inflammation, altered immunometabolism, and macrophage-associated inflammatory crosstalk (47, 163). However, these patterns should currently be regarded as provisional mechanistic enrichments rather than fixed subtype definitions. Accordingly, biomarkers discussed in this context should be considered candidate rather than clinically established stratification tools. Markers related to mechanotransduction, innate immune activation, inflammatory mediator expression, or metabolic inflammation may help guide future studies, but their reproducibility, specificity, and predictive value remain to be validated. Future progress will require integrated analysis of human tissue profiling, synovial or circulating biomarkers, imaging features, and longitudinal clinical outcomes to determine whether NF-κB-associated patterns can support meaningful patient stratification or therapeutic selection in OA.

### 8.2 Open questions and future research directions

Despite significant progress, several fundamental questions remain unresolved and warrant focused investigation. First, the concept of a mechanical stress “threshold model” in OA remains incompletely understood. While excessive mechanical loading clearly promotes NF-κB activation, inflammasome signaling, and tissue degeneration, moderate mechanical stress appears to exert protective effects, including suppression of ferroptosis. The molecular determinants that define this threshold—such as signal amplitude, frequency, duration, and cellular adaptation capacity—remain to be elucidated. Resolving this question will be critical for optimizing rehabilitation strategies and physical interventions that aim to restore physiological mechanotransduction without exacerbating inflammation. Second, the cell-type specificity of STING–NF-κB coupling in the osteoarthritic joint requires clarification. Emerging evidence suggests that STING activation may elicit distinct downstream programs in chondrocytes versus synovial macrophages or fibroblasts. Understanding whether STING preferentially drives interferon responses, NF-κB–dependent inflammation, or both in different cellular contexts will have direct implications for the safety and efficacy of STING-targeted therapies in OA. Third, the mechanisms by which NF-κB signaling directs divergent chondrocyte fate decisions—senescence, apoptosis, or ferroptosis—remain poorly defined. Identifying the factors that bias NF-κB signaling toward specific cell death or survival programs, including mitochondrial status, redox balance, metabolic state, and epigenetic landscape, will be essential for designing interventions that preserve cartilage viability while suppressing inflammation. Finally, defining optimal windows and combinations for therapeutic intervention represents a major challenge. Given the extensive crosstalk between NF-κB and pathways regulating oxidative stress, fibrosis, immune polarization, and metabolism, combination therapies may offer superior efficacy compared with single-agent approaches. However, the timing, dosage, and sequence of such interventions must be carefully optimized to avoid unintended suppression of protective NF-κB functions. Future studies integrating longitudinal biomarker profiling with mechanistic modeling may help identify rational combination strategies tailored to disease stage and subtype.

### 8.3 Single-cell and spatial multi-omics frontiers in decoding NF-κB signaling heterogeneity in osteoarthritis

A major limitation of current OA research is that much of the evidence for NF-κB activation is derived from bulk tissue or population-averaged assays, which cannot fully resolve the cellular and spatial heterogeneity of the osteoarthritic joint. Bulk transcriptomic, proteomic, or immunoblot-based analyses are highly informative for identifying pathway-associated changes at the tissue level, but they inevitably average signals across mixed cell populations and anatomical compartments (164–166). As a result, they cannot determine whether NF-κB activation arises predominantly from chondrocytes, synovial fibroblasts, macrophages, osteoclast-lineage cells, or rare stress-responsive subpopulations. Nor can they adequately distinguish whether pathway activation is widespread or restricted to spatially defined inflammatory, fibrotic, or mechanically stressed niches. This limitation is particularly relevant in OA, where disease progression reflects multicompartmental and cell-state-specific signaling rather than uniform tissue-wide activation.

In this context, single-cell multi-omics offers an important opportunity to refine the interpretation of NF-κB signaling. Single-cell transcriptomic analyses can identify heterogeneous chondrocyte, fibroblast, macrophage, and bone-associated cell states, including inflammatory, catabolic, senescent, reparative, and stress-adapted populations that may differ substantially in their NF-κB-associated transcriptional programs (164, 166). Single-cell epigenomic approaches, including chromatin accessibility profiling, can further reveal whether NF-κB target engagement depends on cell-state-specific regulatory landscapes rather than simply on pathway exposure. Integrative single-cell multiome strategies are especially valuable because they connect transcriptional output with regulatory potential, helping to explain why apparently similar upstream stimuli may produce divergent downstream responses in distinct cellular subsets (73, 167). Such approaches are well suited to address one of the central unresolved questions in OA biology: how NF-κB signaling is differentially encoded and decoded across heterogeneous cell populations within the same diseased joint.

Spatially resolved multi-omics adds an additional layer of insight by preserving tissue architecture and microenvironmental context. Spatial transcriptomics, multiplex imaging, and spatial proteomic approaches can identify localized zones of inflammatory signaling, matrix remodeling, fibrosis, and immune-cell accumulation that are not evident in dissociated-cell datasets (168–170). This is particularly important for understanding NF-κB signaling, because pathway activation in OA is likely to be conditioned not only by cell identity but also by anatomical location, local mechanical load, DAMP concentration, senescence burden, cytokine gradients, and cell–cell interactions. For example, inflammatory activation at cartilage–synovium interfaces, SASP-enriched stromal niches, macrophage-rich synovial regions, or remodeling-associated subchondral bone microenvironments may each impose distinct constraints on NF-κB pathway engagement. In this sense, spatial technologies help reveal microenvironmental conditioning that remains effectively invisible to bulk assays.

Looking forward, the greatest value of these technological advances may lie in integrative analysis rather than in any single platform alone. Combining single-cell transcriptomics, epigenomics, spatial mapping, and signaling-state readouts has the potential to define cell-type-specific NF-κB engagement, identify pathogenic communication circuits, and distinguish driver from bystander inflammatory states across OA progression. Such approaches may also improve mechanistic stratification by identifying NF-κB-dependent OA subtypes, early disease-associated cell states, and spatially restricted therapeutic vulnerabilities. Therefore, single-cell and spatial multi-omics should not be viewed merely as descriptive technologies, but as essential tools for building a more precise and context-aware model of NF-κB signaling in osteoarthritis.

In summary, positioning NF-κB at the center of OA pathogenesis provides a unifying framework that links mechanical stress, innate immunity, metabolic inflammation, and tissue degeneration. Moving forward, the challenge lies in translating this systems-level understanding into clinically actionable strategies that embrace disease heterogeneity rather than obscure it. By integrating mechanistic stratification, biomarker development, and network-informed therapeutic design, the field can move closer to precision medicine approaches capable of restoring joint homeostasis in osteoarthritis.

### 8.4 Emerging trends, therapeutic opportunities, and technological frontiers

Several emerging trends are beginning to reshape how NF-κB signaling is interpreted in osteoarthritis. First, the field is moving away from viewing NF-κB as a linear inflammatory pathway and toward understanding it as a context-dependent signaling hub that integrates mechanical stress, innate immune activation, metabolic state, mitochondrial signaling, and aging-related inflammatory priming (35, 171). Second, there is a growing shift from bulk tissue descriptions toward cell-state- and niche-resolved models of disease, reflecting the recognition that NF-κB signaling is neither spatially uniform nor functionally equivalent across joint compartments. Third, therapeutic thinking is evolving from indiscriminate pathway inhibition toward context-aware modulation, with increasing emphasis on preserving adaptive stress responses while preventing persistent pathological signaling. Together, these trends suggest that future progress in OA research will depend less on identifying single dominant pathways and more on defining how signaling networks are differentially engaged across disease subtypes and tissue microenvironments.

These conceptual shifts also expand the spectrum of potential therapeutic targets. In addition to directly targeting NF-κB core signaling, future strategies may benefit from focusing on upstream mechanosensitive and innate immune nodes, including Piezo1, TRPV4-associated structural gating, TRPM2, cGAS–STING, and DAMP–TLR pathways (36, 54, 55). Equally important are regulatory nodes that shape pathway persistence and context, such as mitochondrial quality control, redox-buffering systems, the NAD^+^/Sirt1 axis, and senescence-associated inflammatory amplification. At the tissue level, macrophage polarization programs, chemokine circuits, fibroinflammatory remodeling, and SASP-enriched degenerative niches may also represent therapeutically actionable NF-κB-associated microenvironmental modules. Rather than broad pathway blockade, such approaches may allow more selective attenuation of pathogenic NF-κB activity while minimizing disruption of homeostatic signaling.

Technological advances will be essential for addressing the major gaps that currently limit the field. Single-cell transcriptomics and multiome platforms can help resolve cell-type-specific NF-κB-associated programs that remain obscured in bulk assays. Spatial transcriptomics, multiplex imaging, and spatial proteomics can identify localized inflammatory, fibrotic, and senescence-associated niches in which NF-κB signaling is conditioned by tissue architecture and cell–cell interactions. Functional phospho-signaling profiling and perturbation-based approaches may be particularly important for distinguishing pathway presence from pathway activity and for clarifying causal rather than correlative signaling relationships. More broadly, integration of single-cell, spatial, epigenomic, and signaling-state data may enable the identification of NF-κB-dependent OA subtypes, early pathogenic cell states, and anatomically restricted therapeutic vulnerabilities. These technological frontiers are therefore not merely methodological refinements, but central tools for translating current mechanistic insights into more precise and clinically actionable models of osteoarthritis.

### 8.5 Field controversies, methodological limitations, and limitations of the present review

Despite the substantial progress made in understanding NF-κB signaling in osteoarthritis, several important controversies and methodological limitations continue to constrain the field. First, much of the current literature establishes association rather than causation. NF-κB activation is frequently observed in parallel with cartilage degeneration, synovitis, immune remodeling, and subchondral bone changes, but it is often unclear whether NF-κB functions as a primary disease driver, a signal amplifier, or a secondary response to pre-existing tissue injury. This distinction is especially important in OA, where mechanical damage, cellular stress, innate immune activation, and aging-related degeneration are tightly intertwined.

Second, commonly used experimental models only partially capture the complexity of human OA. *In vitro* systems based on isolated chondrocytes stimulated with IL-1β or TNF are useful for mechanistic interrogation but may overrepresent acute inflammatory signaling while underrepresenting multicellular crosstalk, chronicity, and biomechanical context (36, 171). Likewise, surgically induced or chemically induced animal models reproduce selected aspects of joint degeneration but do not fully reflect the heterogeneity, aging dependence, metabolic comorbidity, and slow progression of human disease. As a result, findings derived from reductionist models should be interpreted with caution when extrapolating to clinical OA.

Third, pharmacological interpretation remains challenging. Many compounds described as NF-κB inhibitors are not pathway-specific and may act through upstream kinases, redox-sensitive signaling, mitochondrial stress responses, or broader anti-inflammatory mechanisms (172, 173). Consequently, suppression of NF-κB-associated readouts, such as p65 phosphorylation or cytokine expression, does not necessarily establish that therapeutic benefit is mediated specifically through NF-κB blockade. Relatedly, the literature remains heavily weighted toward canonical NF-κB signaling, whereas the contribution of non-canonical pathway activity is still comparatively underexplored and, in some contexts, contradictory. This imbalance in evidence should be considered when drawing mechanistic conclusions. A further limitation of the field is the continued reliance on bulk or population-averaged assays. Such approaches are highly informative at the tissue level, but they may obscure rare pathogenic subpopulations, cell-state-specific pathway engagement, and spatially restricted inflammatory niches. Given the increasing recognition that OA is a multicompartmental and heterogeneous disease, more refined cell-type-resolved and spatially informed approaches will be necessary to define how NF-κB signaling operates across different joint microenvironments and disease stages.

The present review also has several limitations. First, it is intentionally organized around an NF-κB-centered framework and therefore does not aim to provide an exhaustive overview of all signaling pathways involved in OA pathogenesis. This conceptual focus may privilege NF-κB-associated mechanisms over parallel or NF-κB-independent processes that also contribute to disease progression. Second, the evidence base discussed here is uneven in depth and maturity. Some areas, such as canonical inflammatory signaling, cartilage catabolism, and macrophage-associated mechanisms, are supported by a relatively broader literature, whereas other themes—including non-canonical NF-κB signaling, NAD^+^-dependent regulation, spatially resolved pathway activity, and single-cell decoding of NF-κB dynamics—remain emerging and less definitively established. Third, many of the translational implications discussed in this review are still grounded primarily in preclinical evidence, and their clinical relevance remains to be validated in human stratified studies.

Taken together, these limitations do not diminish the importance of NF-κB in OA, but they do argue for a more cautious and evidence-weighted interpretation of the field. A more precise understanding of causality, cell-state specificity, pharmacological selectivity, and disease heterogeneity will be essential for translating NF-κB-centered insights into clinically actionable strategies.

## 9 Conclusion

In conclusion, accumulating evidence positions NF-κB as a central signaling hub that integrates mechanical stress and innate immune inputs to determine the trajectory of osteoarthritis progression. Rather than functioning as a linear inflammatory pathway, NF-κB operates as a dynamic network integrator that translates mechanotransduction, ion channel activity, mitochondrial stress, DNA sensing, and cytokine signaling into coordinated transcriptional programs. Through this integrative role, NF-κB governs extracellular matrix degradation, chondrocyte fate decisions, synovial inflammation and fibrosis, immune cell polarization, and subchondral bone remodeling, thereby orchestrating joint-wide degeneration. Importantly, the pathological impact of NF-κB signaling is highly context dependent, shaped by the source, intensity, and duration of upstream stimuli as well as the metabolic and aging state of joint cells. These insights underscore the limitations of indiscriminate NF-κB inhibition and instead highlight the need for mechanism-informed therapeutic strategies. Future progress in OA management will depend on precise disease stratification based on dominant NF-κB–activating drivers and on rational combination therapies that modulate NF-κB-associated networks rather than single nodes. By embracing this hub-centered and subtype-aware framework, it may be possible to shift OA treatment from symptomatic control toward restoration of joint homeostasis.
